# Artificial exosomes mediated spatiotemporal-resolved and targeted delivery of epigenetic inhibitors

**DOI:** 10.1186/s12951-021-01107-9

**Published:** 2021-11-17

**Authors:** Huan Li, Songpei Li, Yinshan Lin, Sheng Chen, Langyu Yang, Xin Huang, Hao Wang, Xiyong Yu, Lingmin Zhang

**Affiliations:** 1 Key Laboratory of Molecular Target and Clinical Pharmacology and the State and NMPA Key Laboratory of Respiratory Disease, School of Pharmaceutical Sciences and The Fifth Affiliated Hospital, Guangzhou Medical University, Guangzhou, 511436 People’s Republic of China; 2 Department of Oncology, The Sixth Affiliated Hospital of Guangzhou Medical University, Qingyuan People’s Hospital, Qingyuan, 511518 Guangdong China

**Keywords:** Artificial exosomes, Upconversion nanoparticles, Epigenetic inhibition, M1 macrophages, Spatiotemporal-resolved delivery

## Abstract

**Background:**

Malignant tumor is usually associated with epigenetic dysregulation, such as overexpression of histone deacetylase (HDAC), thus HDAC has emerged as a therapeutic target for cancer. Histone deacetylase inhibitor has been approved for clinical use to treat hematological cancers. However, the low solubility, short circulation lifetime, and high cytotoxicity partially limited their applications in solid tumor.

**Methods:**

The upconversion nanoparticles (UC) modified with mesoporous silica (SUC) was used to load an HDACI, suberoylanilide hydroxamic acid (SAHA), and further camouflaged with M1 macrophage-derived exosome membranes (EMS). EMS was characterized in size and compositions. We also analyzed the epigenetic regulation induced by EMS. Furthermore, we evaluate the biodistribution and in vivo tumor inhibition after the systemic administration of EMS.

**Results:**

This novel style spatiotemporal-resolved drug delivery system, EMS showed a high loading efficiency of SAHA. EMS could be taken up by lung cancer cells and lead to efficient epigenetic inhibition. We found that the integrin α4β1 on M1-EM, was crucial for the homing of EMS to tumor tissues for the first time. In tumor-bearing mice, EMS showed spatiotemporal-resolved properties and facilitated the drug accumulation in the tumors, which induced superior anti-tumor effects.

**Conclusion:**

This novel style of spatiotemporal-resolved nanoparticles can be used as a theranostic platform for lung cancer therapy.

**Graphical Abstract:**

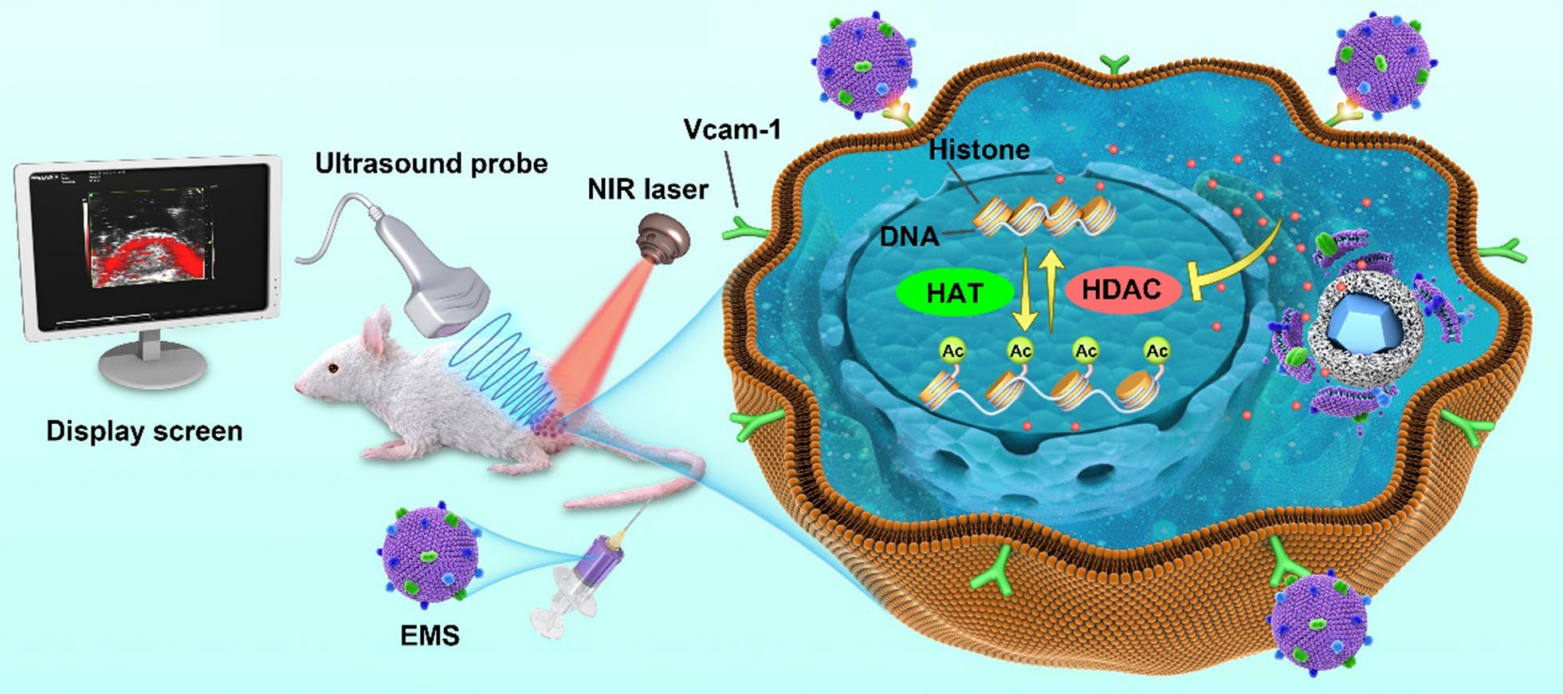

**Supplementary Information:**

The online version contains supplementary material available at 10.1186/s12951-021-01107-9.

## Background

Cancer remains a global public health problem that accounts for about 1 in 6 deaths in the world according to the World Cancer Report by WHO. It has been well recognized that malignant tumor is associated with epigenetic dysregulation such as overexpression of histone deacetylase (HDAC) [1]. Thus, HDAC has been developed into a therapeutic target for cancer [2–4]. So far, several HDAC inhibitors have been applied in human to treat hematologic cancers. Suberoylanilide hydroxamic acid (SAHA) is an FDA-approved HDAC inhibitor for the treatment of cutaneous lymphomas. However, SAHA has low solubility in water, short circulation lifetime, and high cytotoxicity, which may partially account for its failure to treat solid tumors [5]. Previous reports indicated that the fabrication of SAHA prodrug or encapsulation of SAHA into nanoparticles might enhance its therapeutic effect on solid tumors [5].

As the advances in nanotechnology, multifunctional nanoparticles have emerged as theranostic platforms for cancer therapy. These nanoparticles provide noninvasive molecular imaging to track disease stages and biodistribution of the drug in real-time [6]. Lanthanide-doped upconversion nanoparticles (UCs) can be used as fluorescence probes for tumor diagnosis [7] and nano transducers for photodynamic therapy of deep tumors by converting near-infrared radiation to visible light [8]. Photoacoustic (PA) imaging is a high-resolution imaging system, integrating the high selectivity of optical imaging and the penetration depth of ultrasonic imaging. UCs can be used as a contrast agent for PA imaging due to their plasmonic nanostructures [9]. Coating of mesoporous silica on the surface of UCs confers superior load efficiency for drug delivery [10].

Cell membrane-cloaked biomimetic nanoparticles have emerged as promising drug delivery systems for cancer treatment [11]. Various types of cell membrane have been utilized to coat nanoparticles, such as blood cells including red blood cells [12], neutrophils [13], and platelets [14], stem cells [15], and cancer cells [11]. These cell membrane coating either assists the nanoparticles in escaping from immune recognition or confers homotypic targeting capacity to nanoparticles. Macrophages play an important role in regulating the development and metastasis of cancer, of which M1 macrophages can suppress tumors by activating tumor-killing mechanisms [16]. M1 macrophages have a long-circulating half-life and showed tumor-targeting capacity [17]. Thus, M1 macrophages have been investigated as carriers for targeted drug delivery to cancer [17]. However, the cytotoxicity of anti-cancer drugs towards live macrophages may hinder this application of macrophages. Also, the polarization of macrophages is a dynamic process that is regulated by the microenvironment. Live M1 macrophage may be reprogrammed into M2 phenotype in living systems.

Exosomes are membrane-bound extracellular vehicles generated by cell membrane invasion [18]. This natural-occurring nanovesicle has been demonstrated to be a superior drug delivery system with high loading efficacy and can decrease the adverse effects of doxorubicin (DOX) on the heart [19]. Inspired by the recent advances in cell-membrane coated nanoparticles, the exosome membrane has been utilized to decorate nanoparticles [20]. Interestingly, a recent report indicated that cancer cell-derived exosome membrane coating showed better preformation in immune evasion and tumor targeting than the cancer cell membrane [21]. M1 macrophage-derived exosome may possess additional advantages, such as the accessibility from the induction of autologous monocyte, which reduced the immunogenicity significantly; the avoidance of using cancer cell components; the extension of circulation lifetime. Besides, considering that the exosome membrane has similarities with the plasma membrane in protein compositions, we hypothesized M1 macrophages-derived exosomes might inherit the capacity of tumor recognition from M1 macrophage. To our best knowledge, there are rare reports on the application of M1 macrophage-derived exosomes to decorate the nanoparticles for tumor-specific delivery, not to mention the mechanism of action for this style of exosomes.

**Scheme 1:**
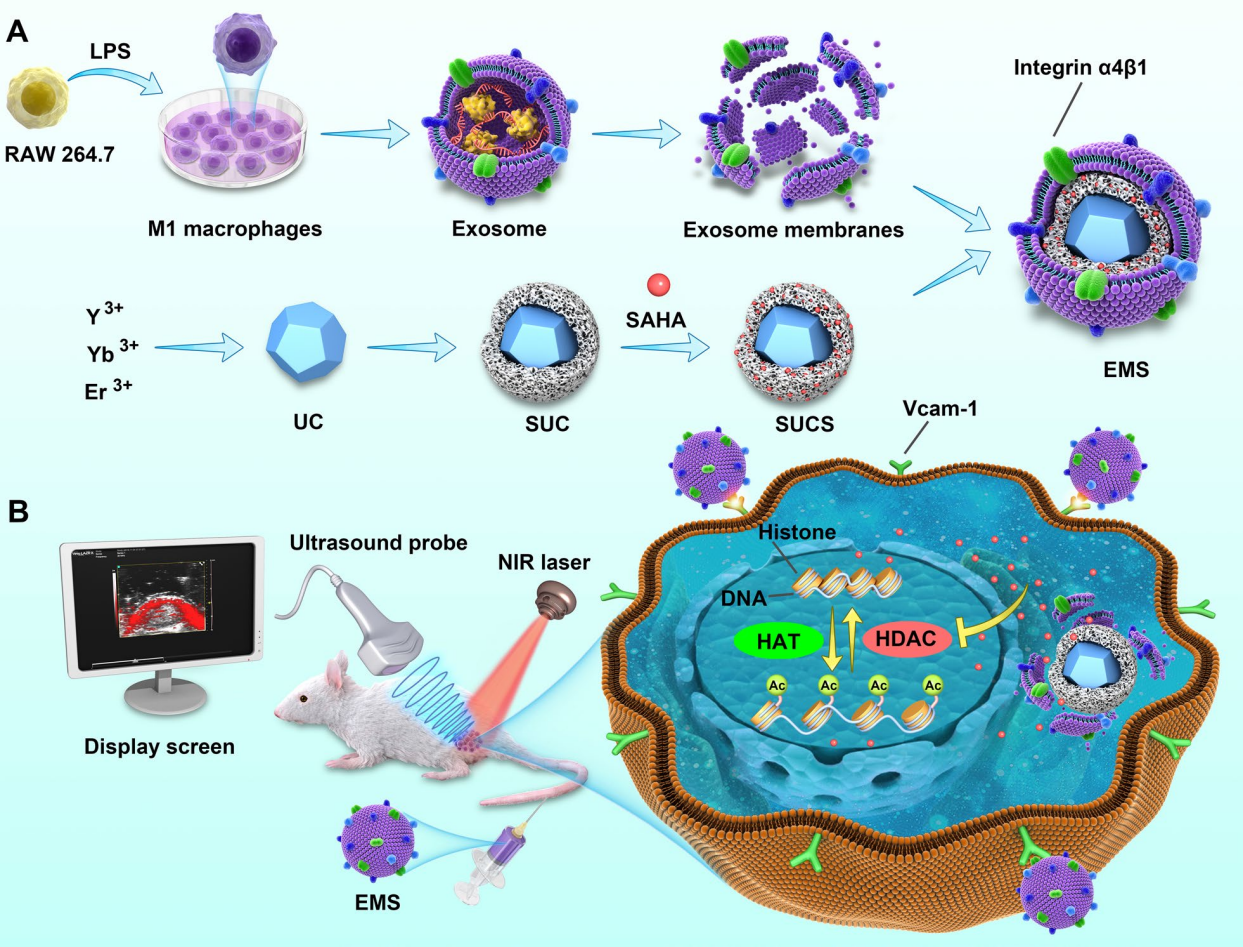
Scheme illustrations of EMS and its applications. **A** The preparation of EMS. **B** The application and biological effects of EMS in vivo. *HAT* histone acetyltransferase, *HDAC* histone deacetylase

Herein, we synthesized mesoporous silica modified β-NaYF4:Er^3+^,Yb^3+^ UCs (SUC), which were used as nanocarriers for the delivery of HDACI, SAHA. SUCs showed an extremely high loading efficiency for SAHA. The M1 macrophage-derived exosome membranes was further used to coat the SAHA-loaded UCs (EMS) (Scheme 1). Interestingly, we found that the EM-camouflaged nanoparticles showed high level of cellular uptake and integrin α4β1 played an important role in mediating the cellular uptake. EMS induced significant histone acetylation, which induced the apoptosis of cancer cells. The in vivo evaluation indicated that the nanoparticles camouflaged with EM enhanced the circulation lifetime. Meanwhile, EMS possessed spatiotemporal-resolved and targeted properties, which was useful in tracking of biodistribution of drugs and improved the drug accumulations to the tumor tissues. EMS also enabled to inhibit the tumor growth significantly. This kind of artificial exosomes provides great potential in ameliorating the effect of epigenetic inhibitors in the solid tumors.

## Materials and methods

### Synthesis of mesoporous silica-coated β-NaYF_4_:Yb^3+^/Er^3+^ nanoparticles (SUC)

β-NaYF_4_:20%Yb, 2%Er UCs were synthesized as previously described with minor modifications [22]. Briefly, oleic acid (8 mL) and 1-octadecene (15 mL) was mixed. RECl_3_ (0.2 M, RE = Y, Yb, and Er (molar ratio = 69:30:1), dissolved in methanol) in a volume of 5 mL was added to the mixture. The mixtures were heated to 160 °C in the argon atmosphere. The temperature was cooled down to 65 °C after the methanol was evaporated. NH_4_F (1.6 M) and NaOH (1.2 M) in methanol (10 mL) were added to the reaction, followed by heating to 300 °C in the argon atmosphere. The reaction continued for 1.5 h. We collected the products by centrifugation after they were cooled down to room temperature. The methanol and cyclohexane were used to wash the resulting products and dried at 60 °C.

The modification with mesoporous silica was performed according to previous work [23]. UCs (7.5 mL) was mixed with a solution containing 2 mL of Triton X-100, 2 mL of hexanol, and 0.6 mL distilled water, and the mixture was stirred for 30 min to form a transparent reverse microemulsion. TEOS (30 μL) and 100 μL of 25% NH_3_·H_2_O were added into the microemulsion under vigorous stirring. The mixture was further stirred for 24 h. The nanoparticles were precipitated by adding 10 mL of acetone and washed with ethanol, followed by centrifugation. The obtained precipitation on the bottom was resuspended in 0.4% CTAC (120 mL) and sonicated to form an emulsion. TEOS (160 μL) and then 1 mL of 2.4% l-arginine were added to the emulsion and stirred at 45 °C for 24 h. The synthesized nanoparticles (SUC) were centrifuged down and washed with 75% ethanol, followed by resuspended in 30 mL of ethanol containing 0.6 g of NH_4_NO_3_. The SUC solution was stirred at 45 °C for 6 h to remove CTAC. SAHA was dissolved in 5% DMSO and mixed with SUCs. The mixture was stirred for 24 h at room temperature. The product (SUCS) was collected by centrifugation (30,000 rpm, 15 min) and washed with distilled water.

### Polarization and identification of M1 macrophages

RAW264.7 cells were cultured in Dulbecco’s Modified Eagle’s Medium (DMEM) supplemented with 10% fetal bovine serum (FBS, GIBCO), 100 U/mL penicillin, and 100 U/mL streptomycin (Gibco) in an atmosphere of 5% CO_2_ at 37 °C. Cells were seeded at a density of 2 × 10^5^ cells/well in a six-well plate and treated with LPS (500 ng/mL) for 1, 3, or 5 days to induce the polarization of RAW264.7 cells towards M1 phenotypes. The polarization of RAW264.7 cells was detected by real-time quantitative PCR (qPCR) analysis of the iNOS, TNF-α, IL-4, and IL-12 expression level, Western blotting analysis of CD80, and iNOS, and flow cytometry analysis of F4/80 and CD80.

### Isolation and identification of M1 macrophage-derived exosomes

M1 macrophages were cultured in media containing exosome-free FBS, and a conditioned medium (CM) was collected for the separation of exosomes. CM was submitted at serial centrifugation at 300×*g* for 10 min to remove live cells, 2000×*g* to remove dead cells, 10,000×*g* for 30 min to remove cell debris. The obtained supernatant was collected for the isolation of exosomes by ultracentrifugation at 100,000×*g* at 4 °C for 6 h. The pallet was washed in PBS and collected by ultracentrifugation. Transmission electron microscopy (TEM) (JEOL JMPEGPTMC-1230, Japan) was used to observe the structure of exosomes.

### Preparation and characterization of EMS

M1 macrophage membrane was separated using a cell membrane isolation kit (Beyotime, China) according to the manufacturer's instruction. The exosomes from M1 macrophages were sonicated to obtain the exosome membrane. SUCS was dissolved in water by a vigorous vortex. The biomimetic EMS and MSUCS nanoparticles were prepared according to the optimized conditions in our previous work [14]. Briefly, the EM or cell membrane suspension with the defined mass ratios (EM: SUC = 1:1) was slowly added to SUCS solution. The mixture was continuously vortexed for 10 min and ultrasonicated for 5 min, followed by continuous extrusion 11 times using Miniextruder (Avanti) to facilitate the coating of membrane on SUCS. The product was collected by centrifugation and washed with water.

TEM (JEOL JMPEGPTMC-1230) was used to detect the structure of the synthesized nanoparticles. The size of nanoparticles was measured by dynamic light scattering (Malvern instrument Zetasizer Nano, UK). Energy dispersive X-ray spectroscopy (EDX) was used to analyse the elemental composition of materials.

### Immunogold staining

EMS solution was pipetted on a glow-discharged, carbon-coated, 400-mesh copper grid. The grid was blocked with 5% bovine serum albumin (BSA; Sigma Aldrich) in PBS for 30 min and then incubated with CD63 antibody (ab217345, Abcam plc., USA) for 1 h, followed by washing with PBS. The grid was further incubated with a cocktail of gold conjugated secondaries against mouse IgG (5 nm gold; Sigma-Aldrich Inc., USA) for 1 h. The sample was washed with PBS and then fixed with 1% glutaraldehyde (Sigma Aldrich, USA) in PBS, followed by washing with deionized water. Finally, the sample was stained with vanadium for imaging. Images were obtained by using a Zeiss Libra 120 PLUS EF-TEM.

### Loading and release

SAHA was dissolved in 5% DMSO and mixed with SUCs at a serial of mass ratios: 1:0.4, 1:0.8, 1:1.2, 1:1.6, 1:2 as described above. The mixture was stirred for 24 h at room temperature. The product (SUCS) was collected by centrifugation (30,000 rpm, 15 min) and washed with distilled water. The SAHA concentration in the supernatant was determined by a UV–vis spectrophotometer at 284 nm to calculate the loading efficiency (LE) of SAHA or the loading capability (LA) of SUC according to the formulations as below:

$${\text{LE}} = \left( {{\text{W}}_{{\text{Initial SAHA}}} - {\text{W}}_{{\text{SAHA in the supernatant}}} } \right)/{\text{W}}_{{\text{Initial SAHA}}} \times {1}00\%$$

$${\text{LA}} = \left( {{\text{W}}_{{\text{Initial SAHA}}} - {\text{W}}_{{\text{SAHA in the supernatant}}} } \right)/{\text{W}}_{{{\text{SUC}}}} \times {1}00\%$$

where _initial SAHA_ represents the weight of initial SAHA, W_SAHA in supernatant_ represents the weight of SAHA in the supernatant, and W_SUC_ represents the weight of SUC.

SAHA release from EMS was determined by dialysis method. Briefly, EMS containing 1 mg SAHA was put into a dialysis bag, the cut-off molecular weight of which was 3000 Da. The dialysis bag was fully submerged into PBS (40 mL, pH 7.4) or PBS (40 mL, pH 5.0) and then stirred at 37 °C. At the designated time intervals, 2 mL of sample solution was taken out and replaced with an equal amount of PBS. The SAHA concentration was measured by a UV–vis spectrophotometer at 284 nm.

### Cellular uptake and location of nanoparticles

Cellular uptake and localization were detected by a confocal laser scanning microscope (CLSM) and FACS analysis in LLC or A549 cells. Due to the lack of fluorescence, SAHA was replaced by fluorescein isothiocyanate (FITC) for the synthesis of nanoparticles (EM/SUC/FITC, abbreviated as EMF; the weight of FITC was equal to 0.1% of EM/SUC). LLC or A549 cells were cultured in Dulbecco’s Modified Eagle's Medium (DMEM) supplemented with 10% FBS (GIBCO), 100 units per mL of penicillin, and 100 units per mL of streptomycin (Gibco) in an atmosphere of 5% CO_2_ at 37 °C. For detection of cellular uptake by the CLSM, LLC cells were seeded into confocal dishes at a density of 4 × 10^5^ cells per dish and cultured for 12 h. The culture medium was removed and the cells were incubated with different concentration of EMF (1.8 μg/mL, 7.2 μg/mL, 14.4 μg/mL, 21.6 μg/mL). The fluorescence of cells was monitored at different time points (1 h, 6 h, 12 h, 24 h) by a CLSM. LLC or A549 cells were cultured for 12 h in 6-well plates and then treated under the same conditions as CLSM analysis. FACS was used to quantify the FITC-positive LLC or A549 cells.

### Antibody blocking

LLC cells at a density of 1 × 10^5^ cells per dish were seeded on confocal culture dishes. After incubation for 12 h, the cells were pretreated with Anti-Vcam1 antibody (10 μg/mL) for 30 min at 37 °C, followed by incubation with EMF for another 12 h. CLSM analysis were performed to analyse the cellular uptake.

### Cell viability assay

For the Live/Dead cell staining, LLC or A549 cells were planted in confocal culture dishes at a density of 1 × 10^5^/well and cultured for 12 h. These cells were incubated with PBS, free SAHA at the concentration of 8 μg/mL, or the nanoparticles (SUC, SAHA, SUCS, MSUCS, EMS) containing an equivalent amount of SAHA. Cells in the confocal dished were used for Live/Dead cell staining using an assay kit (Thermo Fisher Scientific Inc., USA).

For the evaluation of apoptosis, LLC or A549 cells were plated in 6-well plates at a density of 1 × 10^5^/well. After the treatment with different formulations, the cells were collected for FACS analysis of apoptosis using an Annexin V-FITC/PI apoptosis detection kit (Thermo Fisher Scientific Inc., USA) according to the manufacturer's instruction.

### Western blotting analysis

LLC or A549 cells were seeded into 6-well plates. After incubation for 24 h, cells were treated with EMS for 8 h. Cells were washed with PBS and incubated for another 16 h before protein extraction. Total proteins were extracted using RIPA Lysis Buffer (Thermo Fisher Scientific Inc., USA). WB analysis was performed to evaluate the expressions of H3K9 or H3K27. H3 and GAPDH were used as control.

### RNA-Seq analysis

LLC cells were seeded into 6-cm culture dishes. After incubation for 24 h, cells were treated with PBS or EMS for 8 h. Cells were washed with PBS and incubated for another 16 h before RNA extraction. Total RNA was extracted using Trizol (Thermo Fisher Scientific Inc., USA). RNA-Seq analysis was performed to evaluate the transcriptomic and genomic changes.

### Filter-aided sample preparation for mass spectrometry

Exosomes or LLC membrane were lysed by SDT buffer containing 4% SDS, 100 mM Tris–HCl (pH 7.6), and sonicated for isolation of proteins. The lysate was denaturalized by heating at 95 °C for 15 min. After centrifuged at 14,000×*g* for 40 min, the supernatant was quantified with the BCA assay (Thermo, USA). Proteins (80 μg) were incubated with 100 mM DTT in boiling water for 5 min and then cooled down to room temperature. Samples were mixed with 200 μL UA buffer (8 M Urea, 150 mM Tris–HCl pH 8.5) and filtered through a 30 kDa centrifugal filter tube twice. Then 100 μL iodoacetamide (100 mM, in UA buffer) was added to block reduced cysteine residues, and the samples were incubated for 30 min in darkness. The filters were washed with 100 μL UA buffer three times and then 100 μL 25 mM NH_4_HCO_3_ buffer for twice. Finally, the protein suspensions were digested with 40 μL 25 mM NH_4_HCO_3_ buffer containing 4 μg trypsin (Gibco, USA) overnight at 37 °C, and peptides were collected from the filtrate.

### Mass spectrometry analysis

The peptide of each sample was desalted on C18 Cartridges, then concentrated by lyophilization and reconstituted in 40 μL of 0.1% (v/v) formic acid. LC–MS/MS analysis was performed on a Q Exactive HF-X mass spectrometer (Thermo Fisher Scientific) that was coupled to Easy LLC (Thermo Fisher Scientific). 2 μg peptide was loaded onto the C18-reversed-phase analytical column (Thermo Fisher Scientific, Acclaim PepMap RSLC 50 um × 15 cm, nano viper, P/N164943) in buffer A (0.1% formic acid) and separated with a linear gradient of buffer B (80% acetonitrile and 0.1% Formic acid) at a flow rate of 300 nL/min. The linear gradient was as follows: 5% buffer B for 5 min, 5–28% buffer B for 90 min, 28–38% buffer B for 15 min, 38–100% buffer B for 5 min, hold in 100% buffer B for 5 min. The top 10 abundant precursor ions were selected from the survey scan (350–1800 m/z) for HCD fragmentation. The M1 scans were acquired at a resolution of 70,000 at m/z 200 with an AGC target of 3e6 and a maxIT of 50 ms. MS2 scans were acquired at a resolution of 17,500 at m/z 200 with an AGC target of 2e5 and a maxIT of 45 ms, and isolation width was 2 m/z. Only ions with a charge state between 2 and 6 and a minimum intensity of 2e3 were selected for fragmentation. Dynamic exclusion for selected ions was 30 s. The normalized collision energy was 27 eV.

### Mass spectrometry data analysis

The MS data were analysed using MaxQuant software version 1.5.5.1. MS data were searched against the Uniprot_MusMusculus_17027_20200226 database, downloaded on 2020/02/26. An initial search was set at a precursor mass window of 6 ppm. The search followed an enzymatic cleavage rule of Trypsin/P and allowed maximal two missed cleavage sites and a mass tolerance of 20 ppm for fragment ions. Carbamidomethylation of cysteines was defined as fixed modification, while protein N-terminal acetylation and methionine oxidation were defined as variable modifications for database searching. The cut-off of the global false discovery rate (FDR) for peptide and protein identification was set to 0.01. Protein abundance was calculated on the basis of the normalized spectral protein intensity (LFQ intensity). Proteins had over a twofold increase, 50% reduction, or the *p* < 0.05 (Student's t-test) were considered to be different.

### Gene ontology (GO) annotation

All protein sequences were aligned to the *Mus musculus* database downloaded from NCBI (NCBI-blast-2.2.28+-win32.exe), with only the top 10 sequences that E-value less than or equal to 1 × e^−3^ were selected for further analysis. Secondly, the GO term (database version: go_201504.obo) of the sequence with top Bit-Score was selected by Blast2GO. Then, the annotation of GO terms to proteins was performed by Blast2GO Command-Line. After the elementary annotation, InterProScan was used to search the EBI database by motif and then add the functional information of motif to proteins to improve annotation. Then further improvement of annotation and connection between GO terms were carried out by ANNEX. Fisher's Exact Test was used to enrich GO terms by comparing the number of differentially expressed proteins and total proteins correlated to GO terms.

### Kyoto encyclopedia of genes and genomes (KEGG) pathway annotation

Pathway analysis was performed using the KEGG database. Fisher's Exact Test was used to identify the significantly enriched pathways by comparing the number of differentially expressed proteins and total proteins correlated to pathways.

### In vivo tracking of nanoparticles

BALB/c nude mice were subcutaneously inoculated with 4 × 10^6^ LLC cells at the right front legs to establish the tumor-bearing mouse model. When tumor size reached 100 ± 10 mm^3^, the mice were randomly arranged into six groups: saline, DiR, SUC/DiR, MM/SUC/DiR, EM/SUC/DiR. DiR was used as a substituent for SAHA. An equal volume (200 μL) of saline, 50 μg/mL DiR and above nanoparticles containing the same amount of DiR and 1 × PBS were injected into the mice from each group via tail vein. The fluorescence and PA imaging of the tumor was monitored at 3 h, 6 h, 12 h, 24 h, and 48 h after administration by using the LAZR-X multimodal imaging system.

### Animal experiments

The male BALB/c nude mice (4 ~ 6-week-old) were purchased from Beijing Vital River Laboratory Animal Technology Co., Ltd. (Beijing, China) and raised in specific pathogen-free environment. All in vivo studies were performed in accordance with the Institutional Authority for Laboratory Animal Care of Guangzhou Medical University (Number, GY2020-046).

### In vivo tumor inhibition

The subcutaneous tumor model of mice was established as described above. When the volume of the tumor reached 100 ± 10 mm^3^, the mice were divided into six groups (n = 4): saline, SAHA, SUC, SUCS, MSUCS, EMS. An equal volume (200 μL) of saline, free SAHA (200 μg), and nanoparticles containing the same amount of SAHA were injected into the mice from each group via tail vein every three days for 15 days. The size of the tumor was monitored by measuring its length and width by vernier caliper and calculated as V = (L*W^2^)/2. Tumor volumes were measured every three days. At the end of the study, the mice were sacrificed for the collection of organs and tumors.

Tumor tissues were stored in 4% paraformaldehyde and dehydrated using a Leica tissue processor (Leica, German). The dehydrated samples were embedded in paraffin and cut into 4-μm-thick sections. Mayer’s hematoxylin and eosin (H&E) staining were performed according to the standard protocol from the IHC world. TUNEL staining was performed using an assay kit (Thermo Fisher Scientific Inc., USA) according to the manufacturer’s instruction.

### Statistical analysis

The data were represented as mean ± SD. One-way analysis of variance was used to compare multiple groups, followed by a Tukey’s multiple comparisons test. Student’s *t* test was used for two-group comparisons. Differences at *p* < 0.05 were considered statistically significant.

## Results

### Characterizations of UC-based nanoparticles

The commercial UCs were coated by mesoporous silica (SUC), and the synthesized SUC showed fluorescence with typical peaks at 550 and 650 nm (Additional file 4: Fig. S1). Lipopolysaccharide (LPS) was used to stimulate RAW 264.7 cells. The LPS treated cells showed stellate morphology, a hallmark of macrophage (Additional file 4: Fig. S2) [24]. FACS analysis indicated that ~ 50% of cells were F4/80 and CD80 dual positive on Day 1 and increased to ~ 90% on Day 5 (Additional file 4: Fig. S3). Furthermore, western blotting analysis indicated that LPS treatment significantly increased the expression of M1 macrophage markers, including inducible NO synthase (iNOS) and CD80 (Additional file 4: Fig. S4). The release of typical cytokines of M1 macrophages, including IL-6, TNF-α, and IL-12, had approximately 6 to 600-fold increases than the cells without LPS treatment (Additional file 4: Fig. S5). The exosomes derived from LPS treated RAW264.7 cells (M1-exosomes) were isolated by ultracentrifugation. Transmission electron microscope (TEM) analysis and nanoparticle tracking analysis indicated that the diameter of exosomes was ~ 100 nm (Additional file 4: Fig. S6), which was similar to the ones derived from untreated RAW264.7 cells.

**Fig. 1:**
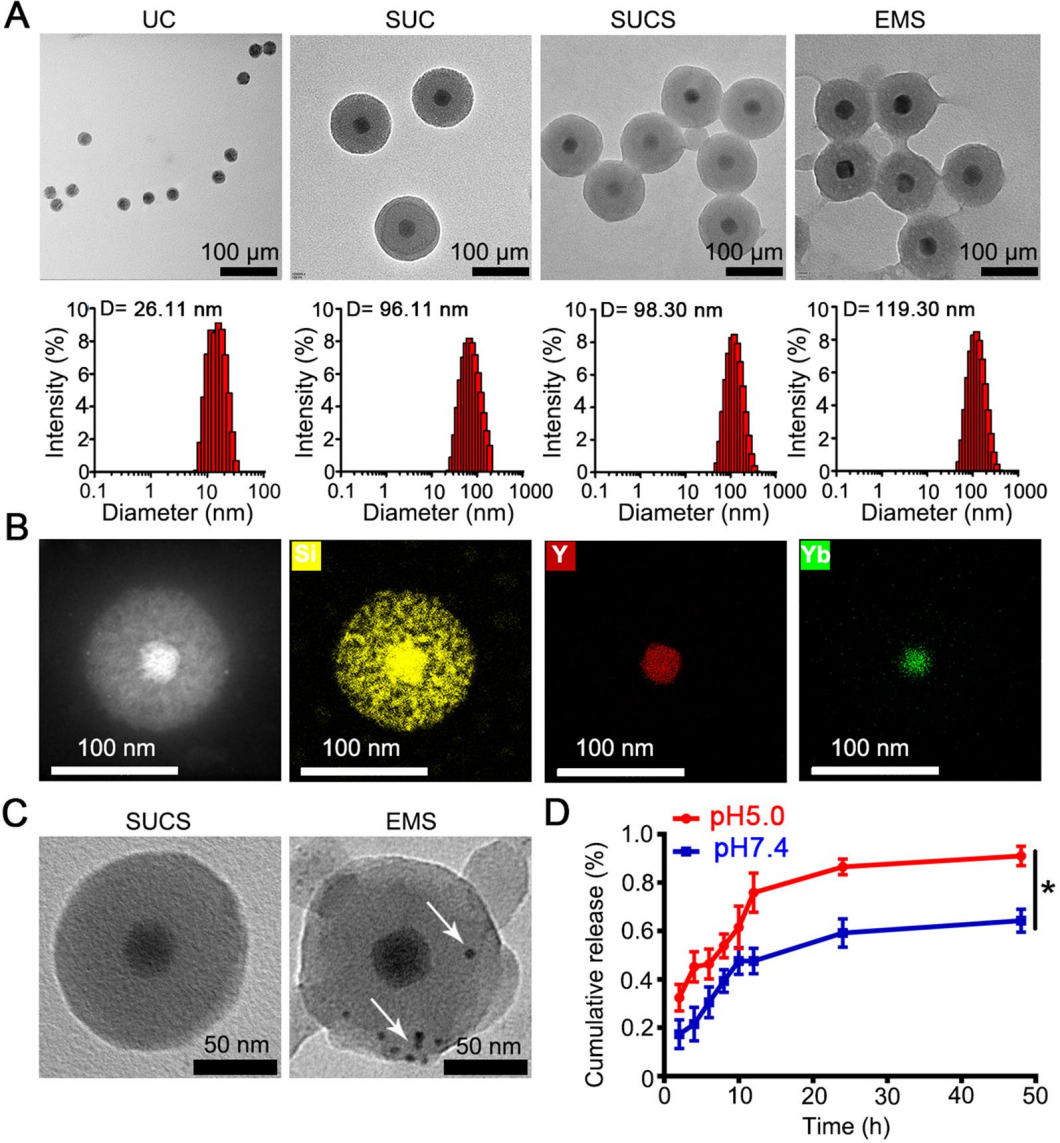
Characterizations of UC-based nanoparticles. **A** TEM and DLS analysis of UC-based nanoparticles. **B** EDX mapping of EMS. **C** Immunogold labeling of CD63 of EMS. **D** Cumulative release of SAHA from EMS in different pHs. The data in **D** are expressed as mean ± SD, n = 3. The comparison was performed with unpaired two-way Student’s *t*-tests. *P < 0.05

To evaluate the loading efficiency of SUC, we used SUC to load SAHA (SUCS) at different concentrations. The results indicated that the loading efficiency was ~ 70% at a SAHA concentration of 1.6 mg/mL (Additional file 4: Fig. S7), with a loading capability of over 113%, which indicated that SUC was an excellent drug delivery vehicle. SUCS was further decorated by M1-EM to confer exosome-mimetic properties to nanoparticles (EMS). TEM, dynamic light scattering (DLS) analysis, and EDX mapping were used to identify the size, multilayer structure of EMS. The TEM analysis revealed that the UCs core with a diameter of ~ 30 nm was encapsulated by a mesoporous silica layer, forming a ~ 100 nm SUC (Fig. 1A). Loading of SAHA and decoration of exosome membrane did not increase the size of nanoparticles according to TEM images. DLS analysis indicated that the median diameter of UCs was 26.11 nm and increased to 96.11 nm after silica coating (Fig. 1A). Similar to TEM observations, SAHA loading did not significantly change the size of nanoparticles. The diameter of EMS increased to 119.30 nm, indicating the success of coating exosome membrane on nanoparticles.

The composition of EMS was further identified by EDX and immunogold staining (Fig. 1B). EDX analysis indicated the presence of Si, Y, and Yb in the EMS. The exosome membrane on EMS was further testified by immunogold labeling of CD63, which is a surface marker of exosomes [25]. The gold nanoparticles with a size of 5 nm were observed on EMS after immunogold labeling (indicated by the white arrows), which is absent in SUCS without exosome membrane coating (Fig. 1C). The release of SAHA from EMS was quicker in a weak acidic environment (pH 5.0) compared with a neutral environment (pH 7.4) (Fig. 1D). This is consistent with the previous report that the acidic environment (pH 5.0) increased the release of DOX from mesoporous silica-coated nanoparticles compared with the neutral environment (pH 7.4) [26]. It might also be explained by the improved hydrophilicity and higher solubility of SAHA in acid conditions, attributed to the increased protonation of -NH groups in SAHA at lower pH.

### Cellular uptake and in vitro tumor cell inhibition

**Fig. 2:**
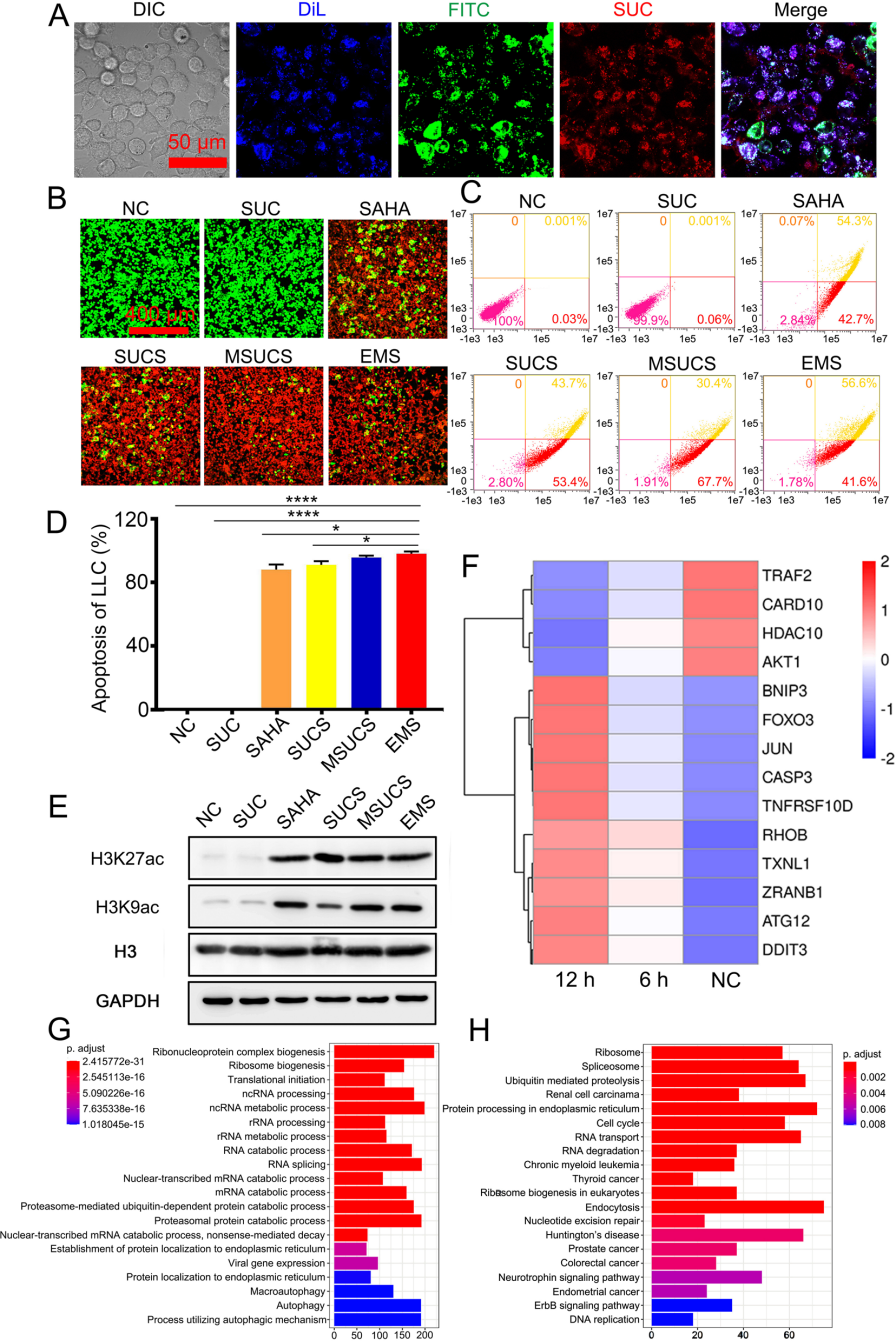
Cellular uptake of artificial exosomes and the nanoparticles induced epigenetic regulation. **A** CLSM analysis of the cellular uptake of EMF. **B** Live/Dead analysis of LLC cells treated with different formulations; **C** Apoptosis of LLC cells treated with different formulations; **D** The quantitative analysis of apoptosis of LLC cells (n = 3); **E** Western blotting analysis of LLC cells treated with different formulations; **F** RNA-Seq analysis of EMS treatment; **G** GO analysis of EMS treatment; **H** KEGG analysis of EMS treatment. The cell membranes were stained with DiL. EMF, EM/SUC/FITC. The data were represented as Mean ± SD. The statistical significance was calculated via one-way ANOVA with a Tukey post-hoc test. *P < 0.05; ****P < 0.0001

The uptake by target cells is a prerequisite for nanoparticles to exert cellular activities. We evaluated the cellular uptake of EMS in a non-small cell lung cancer model, Lewis lung carcinoma (LLC) or A549 cells. A fluorescent dye, FITC was used as a substitution of SAHA (EMF), and exosome membrane was labeled by DiL. Blue (DiL), green (FITC), and red (SUC) fluorescence was observed within LLC cells by CLSM, suggesting that EMF could be taken up by LLC cells (Fig. 2A). The cellular fluorescence increased with the incubation time and concentration of EM-camouflaged nanoparticles and reached a plateau at 12 h (Additional file 4: Fig. S8), and dosage of 14.4 μg/mL (Additional file 4: Fig. S9). The cellular uptake was also performed in the human derived lung cancer cell line A549 cells and the result was similar to the LLC cells (Additional file 4: Figs. S10, S11). Thus, the condition of incubation for 12 h and dosage of 14.4 μg/mL was selected for further cell experiments. The cytotoxicity and apoptosis induced by the nanoparticles were evaluated by Live/Dead cell staining and Annexin V-FITC/PI staining, respectively. Cell viability analysis with cell counting kit-8 (CCK-8) (Additional file 4: Fig. S12) and Live/Dead cell staining (Fig. 2B, Additional file 4: Fig. S13A) showed that SUC alone did not induce significant cell death. In contrast, SAHA alone led to apparent cell death. The incorporation of SAHA into the nanoparticles (SUCS, MUCS, EMS) did not alter the cytotoxicity of SAHA. The Annexin V-FITC/PI staining showed similar results that SAHA and SAHA-containing nanoparticles led to cell death of ~ 95% (Fig. 2C, D; Additional file 4: Fig. S13B, C).

WB and RNA-Seq analysis were performed to evaluate the changes in the protein and mRNA level. WB analysis showed that SAHA per se or SAHA-loaded nanoparticles increased the acetylation of histone significantly, including acetylated histone H3 Lysine 9 (H3K9ac) and histone H3 Lysine 27 (H3K27ac), which are epigenetic marks (Fig. 2E) [27]. Similar results were also found in A549 cells (Additional file 4: Fig. S13D). RNA-Seq analysis indicated that the SAHA-containing EMS reduced HDAC10 gene expression, which is associated with decreased expression of target genes of HDAC, including AKT1 (Fig. 2F) [28]. Besides, EMS altered the expression of apoptosis-related genes, such as the decrease expression of anti-apoptosis genes including TRAF2 [29], CARD10 [30], and the increase of pro-apoptosis gene expression including CASP3, RHOB [31], and FOXO3 [32] (Fig. 2E). These data indicated that the treatment with EMS inhibited the HDAC and led to the apoptosis of tumor cells.

GO enrichment analysis indicated the difference in expression of genes in RNA splicing and processing that were recognized under the regulation of HDAC [33], and the expression of genes in cancer-related metabolic processes such as ribosome biogenesis and ribonucleoprotein complex biogenesis (Fig. 2G) [34, 35]. KEGG pathway analysis indicated the effects of EMS on ribosome-related gene expression (Fig. 2H). Besides, the changes in gene expression of the endocytosis pathway, which may contribute to the uptake of EMS as indicated by previous work [36]. These results suggested that the inhibitory effect of drug-loaded artificial exosomes on tumor cells was associated with the suppression of HDAC by SAHA.

### The evaluation of specificity

**Fig. 3:**
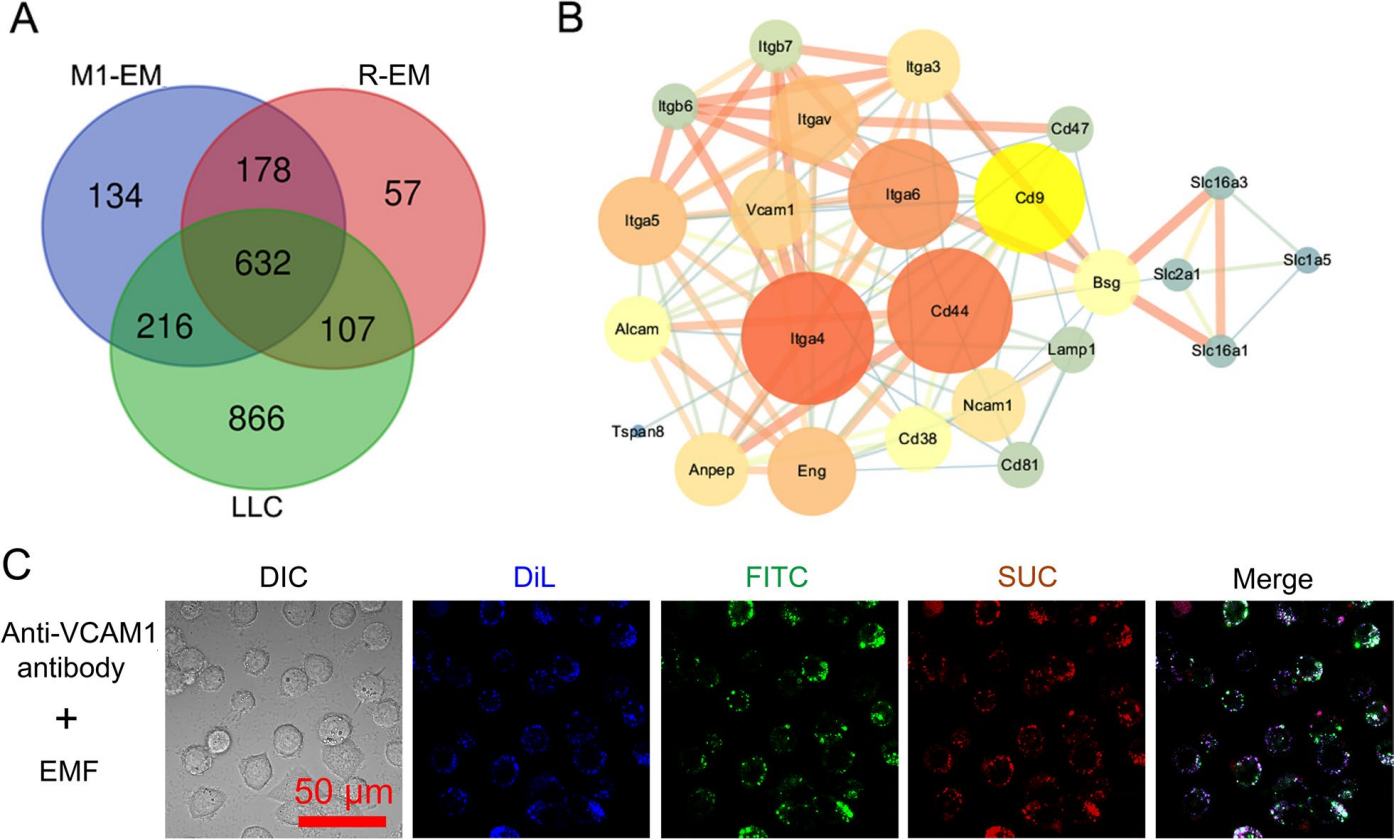
Proteomics analysis of the protein interactions between the membrane proteins of exosomes and LLC. **A** Venn diagram of proteins derived from M1-EM, R-EM, and LLC membrane (n = 3). **B** STRING analysis of the interactions between upregulated proteins (M1-EM vs. R-EM) and LLC cell membranes. **C** The block of cellular uptake of EM-based nanoparticles. The cell membranes were stained with DiL. EMF, EM/SUC/FITC

To investigate the mechanism underlying the improved uptake of exosome membrane camouflaged nanoparticles by LLC cells, quantitative mass spectrometry was used to analyse the protein composition of exosome and LLC membranes, including exosome membrane-derived from M1 macrophages (M1-EM), RAW264.7 cells (R-EM), and LLC cell (Additional file 1: Appendix 1). A total of 1160 proteins were identified in M1-EM, of which 848 proteins were also detected in the LLC membrane. This suggested that M1-EM and LLC membrane possessed a large number of homotypic proteins (73%). In comparison, 739 proteins from R-EM were homotypic to the LLC membrane, which was much less than M1-EM (Fig. 3A). These data indicated that polarized M1 macrophages produced exosomes that have a more similar protein composition to the cancer cell membrane, which may facilitate the homotypic targeting of M1-EM to tumors. Interestingly, we found that 43 proteins were upregulated (Additional file 2: Appendix 2) and 350 proteins were newly created (Additional file 3: Appendix 3) in M1-EM compared with R-EM. To predict the protein interaction between M1-EM and LLC membrane, STRING analysis of these 43 proteins and LLC membrane proteins was performed (Fig. 3B). The results indicated that integrins α4β1 (also named Itga4) has the highest node degree among 43 proteins and interacts with Vcam1, suggesting that this protein may be involved in the interaction between M1-EM and LLC membrane. This is consistent with previous reports indicated that integrin α4β1 medicated the homing and binding of macrophages to metastatic cancer cells [37, 38]. Furthermore, we evaluated the specificity of EM-coumouflaged nanoparticles by the pretreatment of LLC cells with anti-VCAM-1 antibody. CLSM analysis indicated that the cellular uptake reduced ~ 50% (Fig. 3C), compared with the single treatment with EMF (Fig. 2A).

### In vivo tracking of nanoparticles

**Fig. 4:**
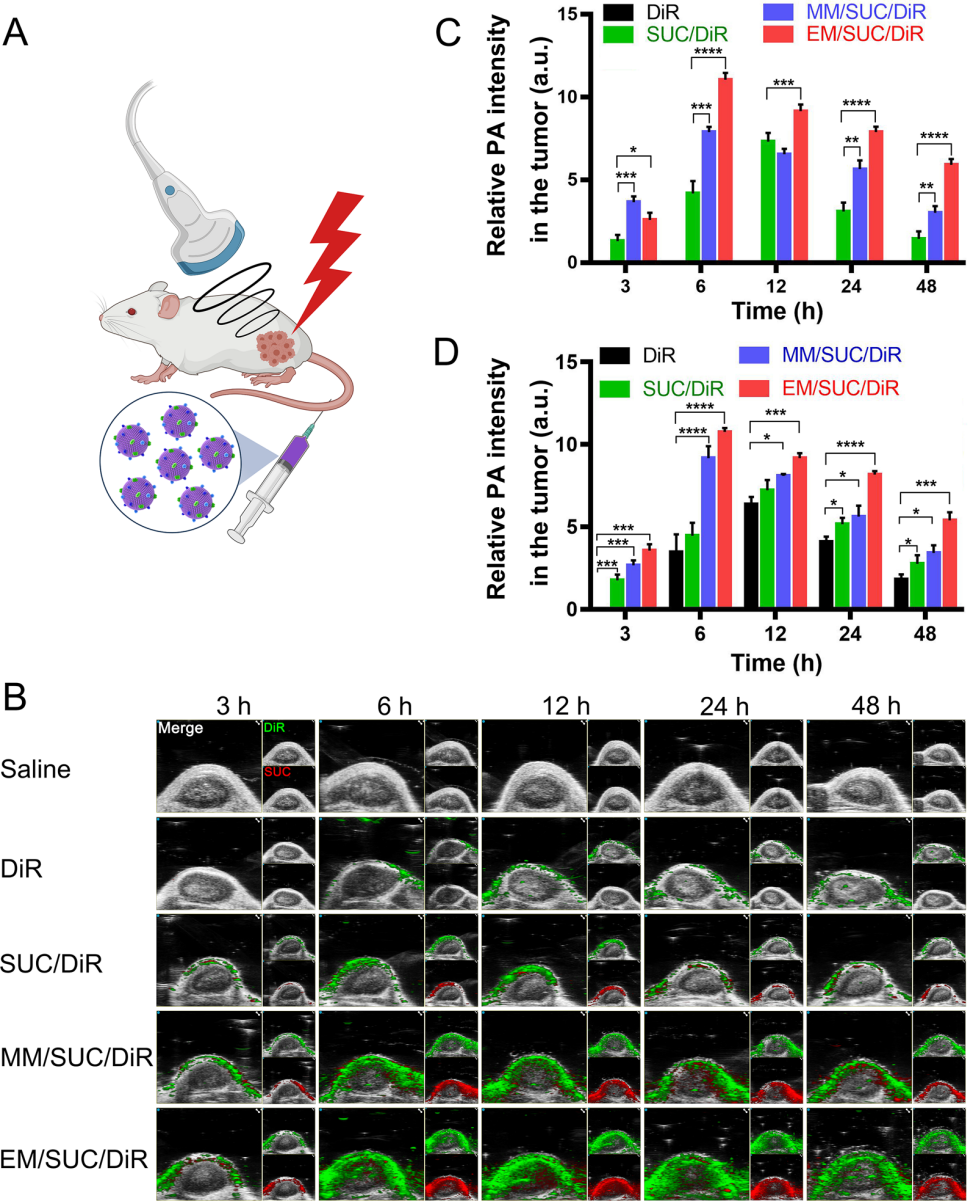
PA analysis of artificial exosomes. **A** Schematic diagram of the administration of SUC-based nanoparticles and the detection of PA signals. **B** PA analysis of the nanoparticles. **C** Quantitative analysis of the signals of SUC. **D** Quantitative analysis of the signals of DiR. EMS were administrated to the mice. PA imaging was used to track the distribution of EMS at different time (3, 6, 12, 24, and 48 h). The quantitative analysis of PA signals was performed with Image J software. The data in **C** and **D** are expressed as mean ± SD, n = 3. The statistical significance was calculated via one-way ANOVA with a Tukey post-hoc test. *P < 0.05; **P < 0.01; ***P < 0.001; ****P < 0.0001

To investigate the distribution of different formulations in vivo, we tracked the nanoparticles by PA imaging (Fig. 4A). DiR was used as a substitute for SAHA and showed a green signal when its fluorescence was detected. The SUC is a superior contrast agent for PA imaging [39]. Obviously, M1-EM decoration increased the accumulation of DiR (green) and SUC (red) in tumor with a considerable amount of DiR delivered to the tumor within 6 h and rationed in tumor 48 h after administration (Fig. 4B). However, the control groups, such as naked DiR and DiR loaded SUC (SUC/DiR), only showed a limited amount of DiR or SUC signals. The coating with M1 macrophage membranes (MM/SUC/DiR) enhanced the accumulation of DiR and SUC within tumor tissues, but the fluorescence intensity was significantly lower compared with M1-EM camouflaged ones (EM/SUC/DiR). The quantitative analysis of SUC (Fig. 4C) or DiR (Fig. 4D) also confirmed the higher accumulations in the tumors compared with the control groups. This can be attributed to the camouflage with M1-EM increased the specificity to the tumor cells as described in proteomics analysis. Furthermore, the camouflage with M1-EM ameliorated the circulation lifetime significantly, compared with bare SUC or M1 macrophage membrane camouflaged nanoparticles (MSUCS) (Additional file 4: Fig. S14). EMS showed spatiotemporal-resolved delivery and facilitated the retention of a drug for tumor-targeted therapy.

### In vivo tumor inhibition

**Fig. 5:**
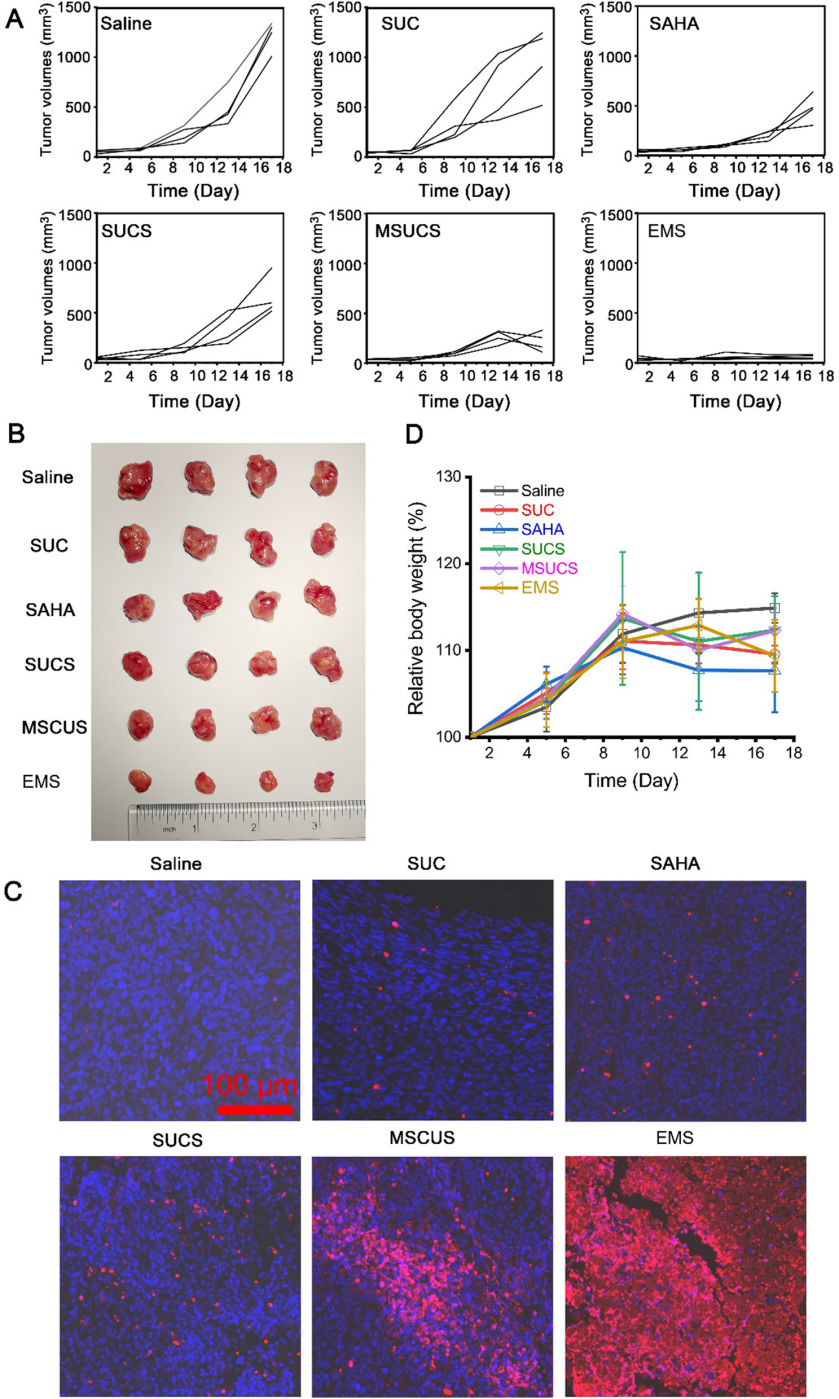
In vivo tumor inhibition. **A** The changes of tumor volumes. **B** The extracted tumors. **C** In situ apoptosis of tumor tissues. **D** The changes of body weight. The data in **D** are expressed as mean ± SD, n = 4. The statistical significance was calculated via one-way ANOVA with a Tukey post-hoc test. *P < 0.05; **P < 0.01; ***P < 0.001; ****P < 0.0001

We further evaluated the tumor inhibition of the synthesized nanoparticles in vivo. The LLC-tumor-bearing mice were constructed by the injection of LLC cells on the right shoulder. After the tumors grew to 100 mm^3^, the mice were administered with control (saline) or different formulations every 3 days, and the size of tumor tissues was monitored. SUC alone did not induce consistent inhibition of tumor growth with 3 mice that had a tumor size over or slightly lower than 1000 mm^3^, and only one mouse had a tumor of ~ 500 mm^3^ at the end of the study (Fig. 5A). SAHA treatment significantly decreased the size of the tumor, the final volume of which was approximately 500 mm^3^. Incorporation into SUC did not obviously alter the effect of SAHA. Decoration of macrophage membrane significantly suppressed tumor growth compared with the bare SUCS. Coating of exosome membrane almost completely inhibited the growth of tumor tissue, suggesting a superior therapeutic effect against the tumor. At the end of the study, tumor tissues were harvested, and the volume of tumors was consistent with the monitored ones (Fig. 5B). We also performed TUNEL staining of the tumor section to investigate the in situ apoptosis. EMS led to significant apoptosis across the whole section of the tumor, suggesting EMS penetrated into tumor tissues to exert cytotoxic effect (Fig. 5C). All formulations did not induce obvious weight loss of mice, suggesting low systemic toxic effects (Fig. 5D and Additional file 4: Fig. S15). The in vivo tumor inhibition evaluation indicated that M1-EM membrane camouflaged nanoparticles ameliorated the therapeutic effect of epigenetic inhibitors, which may provide a novel treatment strategy in the field of cancer therapy.

## Discussion

Macrophages, such as tumor-associated macrophages, play an important role in tumor growth, progression, and invasion [40, 41]. Taking full advantage of the properties of macrophages showed great potential in cancer therapy. M1 macrophages have been investigated as vehicles for drug delivery to the tumor since they have a long-circulating half-life and tumor-targeting capacity [17]. However, the cytotoxicity of anti-tumor drugs towards macrophages, and the risk of pulmonary embolism after systemic administrations may hinder the application of live macrophages as a drug carrier. We supposed that the exosomes derived from M1 macrophages inherited the capacity of tumor recognition from M1 macrophage. However, the use of natural exosomes as drug delivery systems shows some disadvantages, such as the low loading efficiency; the incapability to carry hydrophobic drugs; the single-mode performance. In this study, we constructed a novel style of artificial exosomes with M1 macrophage-derived exosome membranes to coat HDACI-loaded nanoparticles. This style of artificial exosomes showed an extremely high loading capability of over 113%. Furthermore, the drug loading artificial exosomes (EMS) were spatiotemporal-resolved by non-invasive photoacoustic imaging, which acted as a platform for integrated diagnosis and treatment.

The present study showed that EMS inhibited tumor growth significantly (Fig. 5). It is well recognized that the first two steps for the delivery of nanoparticles to a solid tumor are circulation in blood and accumulation in the tumor [42]. The efficiency of these steps will determine the therapeutic effects of nanoparticles. EMS showed improved accumulation in the tumors (Fig. 4), which enhanced the local concentrations of HDACI to overcome the tumors. Based on our study, the accumulation of EMS in tumors may be due to the following characterization of EMS: (1) the protein composition of M1-EM was similar to the tumor cell membrane, which facilitated the homotypic targeting of EMS (Fig. 3A); (2) M1-EM had a higher level of integrin α4β1 compared with R-EM (Additional file 1: Appendix 1), which has been shown to medicate the homing and binding of macrophages to metastatic cancer cells; 3) EMS had longer circulating half-life compared with MUCS and naked nanoparticles (Additional file 4: Fig. S14). Exosomes are nano-sized vesicles secreted by the cells for intercellular communication through the delivery of cargo [43], which exist in various biofluids, including blood, urine, tear, and saliva, in physiological conditions. Thus, it is not surprising that these naturally occurring nanovesicles are biocompatible and stable in blood circulation. However, the detailed mechanism underlying the long circulation properties of exosome membrane coated nanoparticles, such as the membrane proteins or other molecules, needs to be investigated in our future study.

SAHA is the first HDACI approved by the FDA for the treatment of cutaneous manifestations of cutaneous T-cell lymphoma. Although it has been shown that SAHA inhibited various tumor cells in vitro, clinical trials have demonstrated that the lack of efficacy of SAHA in solid tumors such as lung cancer as monotherapy [44]. The pharmacokinetic characterization of SAHA should be considered. Due to the chemical properties of its pharmacophore, SAHA has a substantially short elimination half-life which is typically less than 2 h [45]. This greatly hindered its distribution and retention within tumor tissue, leading to a low concentration under the therapeutic dose in its site of action. Targeted delivery has been proposed as a potential strategy to overcome the poor pharmacokinetics and off-target toxic effects of HDACI [43]. In this study, a biomimetic nanocarrier with high loading efficiency was synthesized for the targeted delivery of SAHA. This novel delivery system significantly enhanced the therapeutic efficacy of SAHA (Fig. 5) and may serve as a theranostic platform for real-time monitoring drug distribution and disease progression.

## Conclusions

In conclusion, we have engineered a novel M1 macrophage-derived exosome membrane camouflaged UCs. In order to increase the loading efficiency of this nanoplatform, UCs were coated by mesoporous silica and used for SAHA loading (SUCS). Furthermore, M1-EM was fused onto the SUCS (EMS). Proteomic analysis indicated that M1-EM had more homotypic proteins with the cancer cell membrane and presented a higher level of integrin α4β1, compared with R-EM. EMS could be taken up by lung cancer cells and inhibited HDAC activity. In tumor xenograft mice, EMS facilitated the accumulation and retention of loaded drugs in tumor tissues. Accordingly, EMS showed superior anti-tumor effects in tumor-bearing mice compared with bare drug or SUCS. Inherited from UCs, EMS could be detected by PA imaging, which may be utilized for real-time diagnosis. Taken together, this theranostic nanoplatform provide a novel strategy for the spatiotemporal-resolved delivery of the anti-cancer drug.

## Supplementary Information

**Additional file 1: Appendix 1.** Proteomics analysis of the exosome membrane proteins on different cell lines. M1-EM, exosomes derived from M1 macrophages; R-EM, exosomes derived from RAW264.7 cells; LLC cell, exosomes derived from LLC cells.

**Additional file 2: Appendix 2.** Proteomics analysis of the upregulated membrane proteins of the exosomes derived from M1 macrophages, compared with the one from RAW264.7 cells. M1-EM, exosomes derived from M1 macrophages; REM, exosomes derived from RAW264.7 cells.

**Additional file 3: Appendix 3.** Proteomics analysis of the newly created membrane proteins of the exosomes derived from M1 macrophages, compared with the one from RAW264.7 cells. M1-EM, exosomes derived from M1 macrophages; REM, exosomes derived from RAW264.7 cells.

**Additional file 4: Figure S1.** The emission spectrum of SUC. **Figure S2.** The morphology of macrophages with or without LPS treatment. The concentration of LPS was 500 nM, and the incubation time was 5 days. **Figure S3.** FACS analysis of the LPS-stimulated macrophages. A) FACS analysis of the M1 macrophages. B) Quantitative analysis of M1 macrophages. RAW264.7 cells were treated with LPS for different time. The data were represented as Mean ± SD. The statistical significance was calculated via one-way ANOVA with a Tukey post-hoc test. *P < 0.05; **P < 0.01; ****P < 0.0001. **Figure S4.** WB analysis of maker of M1 macrophages. RAW264.7 cells were treated with LPS for 5 days. The proteins were extracted and performed with WB. **Figure S5.** Relative RNA expressions. RAW264.7 cells were treated with LPS at a concentration of 500 nM for 5 days. The data are expressed as mean ± SD, n = 3. *p < 0.05, **p < 0.01, and ***p < 0.001, unpaired two-way Student’s *t*-tests. **Figure S6.** Transmission electron microscope (TEM) analysis and Nanoparticle tracking analysis of exosomes derived from RAW264.7 cells and M1 macrophages. M1 macrophages were induced by the stimulation of RAW264.7 cells with LPS at a concentration of 500 nM for 5 days. **Figure S7.** The loading efficiency of SUC at different concentrations of SAHA. SAHA was loaded into SUC at a serial of mass ratios: 1:0.4, 1:0.8, 1:1.2, 1:1.6, and 1:2. The data were represented as Mean ± SD, n = 3. The statistical significance was calculated via one-way ANOVA with a Tukey post-hoc test. *P < 0.05. **Figure S8.** Time-dependent cellular uptake of EMS. LLCs were incubated with EM/SUC/FITC for 1, 6, 12, and 24 h, respectively. **Figure S9.** Dosage-dependent cellular uptake. LLCs were incubated with EM/SUC/FITC at different concentrations (1.8, 7.2, 14.4, and 21.6 μg/mL), respectively. **Figure S10.** Time-dependent cellular uptake of EMS. A549 cells were incubated with EM/SUC/FITC for 1, 6, 12, and 24 h, respectively. **Figure S11.** Dosage-dependent cellular uptake. A549 cells were incubated with EM/SUC/FITC at different concentrations (1.8, 7.2, 14.4, and 21.6 μg/mL), respectively. **Figure S12.** Cell viability after the treatment with different formulations. A) LLC cells. B) A549 cells. The dosage of SAHA was 8 μg/mL. The data was represented as mean ± SD. Statistical significance was calculated via one-way ANOVA with a Tukey post-hoc test. **Figure S13.** Different formulations induced epigenetic regulation. A) Live/Dead analysis of A549 cells treated with different formulations. B) Apoptosis of A549 cells treated with different formulations. C) The quantitative analysis of apoptosis of A549 cells (n = 3). D) Western blotting analysis of A549 cells treated with different formulations. The data in C) was represented as mean ± SD. Statistical significance was calculated via one-way ANOVA with a Tukey post-hoc test. *p < 0.05, ***p < 0.001, ****p < 0.0001. **Figure S14.** Circulation lifetime of different nanoparticles. EMS was labeled with DiD and administrated to the mice through tail intravenous injection. The blood was collected at different time points (0, 1, 4, 8, 12, and 24 h), respectively. The fluorescence intensity was examined with a multifunctional microplate reader. The data was represented as mean ± SD. Statistical significance was calculated via one-way ANOVA with a Tukey post-hoc test. *p < 0.05, **p < 0.01, ****p < 0.0001. **Figure S15.** HE staining of the extracted organs. HE staining of the extracted organs. LLC-bearing mice were administrated with EMS every three days. After 15 days, the mice were sacrificed and the major organs were extracted and stained with HE. Saline, SAHA, SUC, SUCS, or MSUCS were used as control.

## Data Availability

All data generated or analyzed during this study are included in this published article and its additional information.

## Abbreviations

CCK-8: Cell counting kit-8
CLSM: Confocal laser scanning microscope
CM: Conditioned medium
DLS: Dynamic light scattering
DOX: Doxorubicin
EMS: SUCS coated by exosome membrane from M1 macrophages
FITC: Fluorescein isothiocyanate
H3K27ac: Histone H3 Lysine 27
H3K9ac: Acetylated histone H3 Lysine 9
HAT: Histone acetyltransferase
HDAC: Histone deacetylase
iNOS: Inducible NO synthase
LLC: Lewis lung carcinoma
LPS: Lipopolysaccharide
M1-EM: Exosome membrane-derived from M1 macrophages
MSUCS: SUCS coated by M1 macrophage membrane
PA: Photoacoustic
qPCR: Quantitative PCR
R-EM: RAW264.7 cells
SAHA: Suberoylanilide hydroxamic acid
SUC: UC modified with silica
SUCS: SUCs loaded with SAHA
TEM: Transmission electron microscopy
UCs: Upconversion nanoparticles
