# Supplementary material for: Artificial exosomes mediated spatiotemporal-resolved and targeted delivery of epigenetic inhibitors
Source: J Nanobiotechnology. 2021 Nov 17;19:364. doi: 10.1186/s12951-021-01107-9 (PMC8597284; doi:10.1186/s12951-021-01107-9)
Supplement: Supplementary file 4 — Additional file 4: Figure S1. The emission spectrum of SUC. Figure S2. The morphology of macrophages with or without LPS treatment. The concentration of LPS was 500 nM, and the incubation time was 5 days. Figure S3. FACS analysis of the LPS-stimulated macrophages. A) FACS analysis of the M1 macrophages. B) Quantitative analysis of M1 macrophages. RAW264.7 cells were treated with LPS for different time. The data were represented as Mean ± SD. The statistical significance was calculated via one-way ANOVA with a Tukey post-hoc test. *P < 0.05; **P < 0.01; ****P < 0.0001. Figure S4. WB analysis of maker of M1 macrophages. RAW264.7 cells were treated with LPS for 5 days. The proteins were extracted and performed with WB. Figure S5. Relative RNA expressions. RAW264.7 cells were treated with LPS at a concentration of 500 nM for 5 days. The data are expressed as mean ± SD, n = 3. *p < 0.05, **p < 0.01, and ***p < 0.001, unpaired two-way Student’s t-tests. Figure S6. Transmission electron microscope (TEM) analysis and Nanoparticle tracking analysis of exosomes derived from RAW264.7 cells and M1 macrophages. M1 macrophages were induced by the stimulation of RAW264.7 cells with LPS at a concentration of 500 nM for 5 days. Figure S7. The loading efficiency of SUC at different concentrations of SAHA. SAHA was loaded into SUC at a serial of mass ratios: 1:0.4, 1:0.8, 1:1.2, 1:1.6, and 1:2. The data were represented as Mean ± SD, n = 3. The statistical significance was calculated via one-way ANOVA with a Tukey post-hoc test. *P < 0.05. Figure S8. Time-dependent cellular uptake of EMS. LLCs were incubated with EM/SUC/FITC for 1, 6, 12, and 24 h, respectively. Figure S9. Dosage-dependent cellular uptake. LLCs were incubated with EM/SUC/FITC at different concentrations (1.8, 7.2, 14.4, and 21.6 μg/mL), respectively. Figure S10. Time-dependent cellular uptake of EMS. A549 cells were incubated with EM/SUC/FITC for 1, 6, 12, and 24 h, respectively. Figure S11. Dosage-dependent cellular [file 12951_2021_1107_MOESM4_ESM.docx]

**Additional information**

**
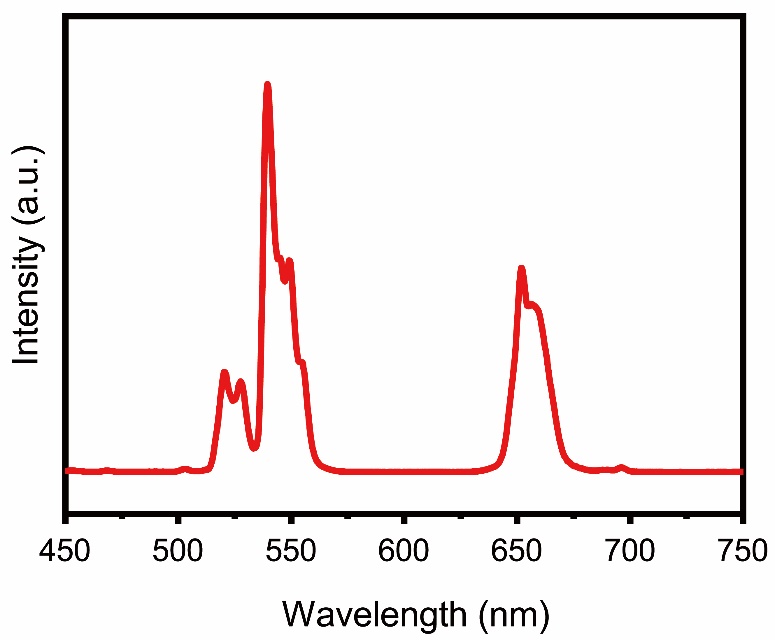
**

**Figure S1.** The emission spectrum of SUC.


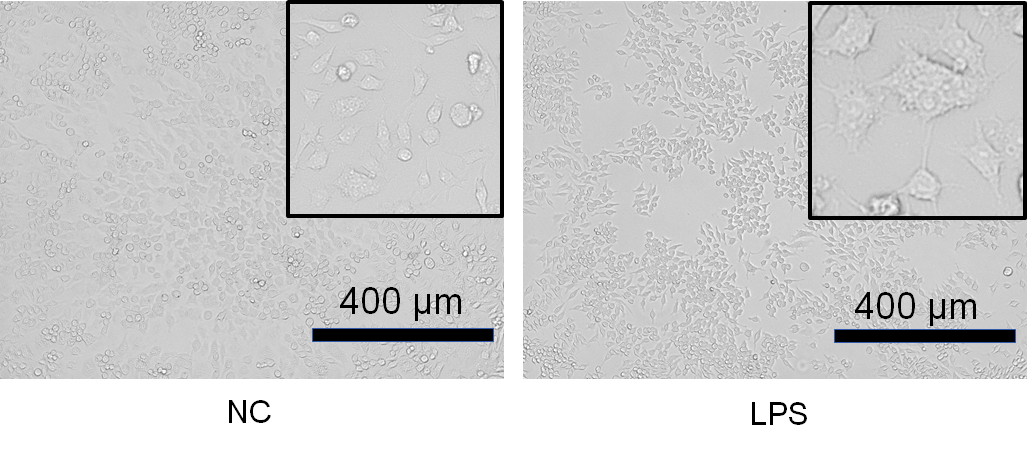


**Figure S2.** The morphology of macrophages with or without LPS treatment. The concentration of LPS was 500 nM, and the incubation time was 5 days.


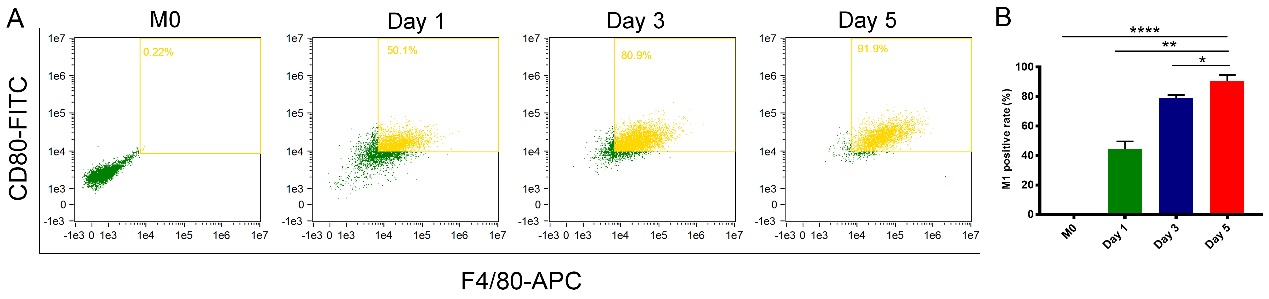


**Figure S3.** FACS analysis of the LPS-stimulated macrophages. A) FACS analysis of the M1 macrophages. B) Quantitative analysis of M1 macrophages. RAW264.7 cells were treated with LPS for different time. The data were represented as Mean ± SD. The statistical significance was calculated via one-way ANOVA with a Tukey post-hoc test. *P < 0.05; **P < 0.01; ****P < 0.0001.


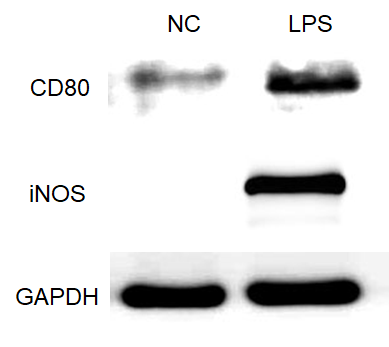


**Figure S4.** WB analysis of maker of M1 macrophages. RAW264.7 cells were treated with LPS for 5 days. The proteins were extracted and performed with WB.


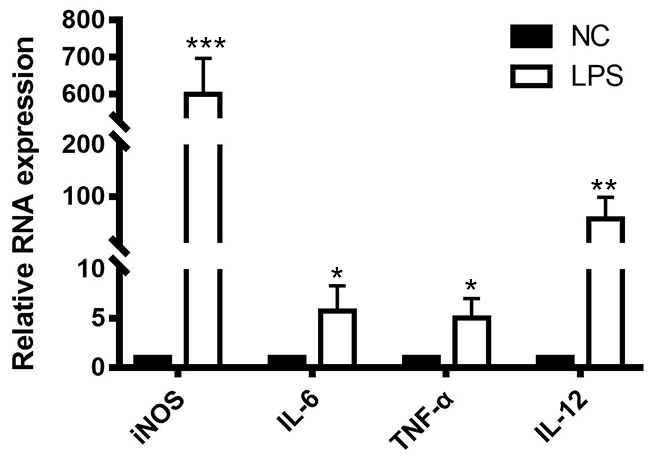


**Figure S5.** Relative RNA expressions. RAW264.7 cells were treated with LPS at a concentration of 500 nM for 5 days. The data are expressed as mean ± SD, n=3. *p<0.05, **p<0.01, and ***p<0.001, unpaired two-way Student’s *t*-tests.


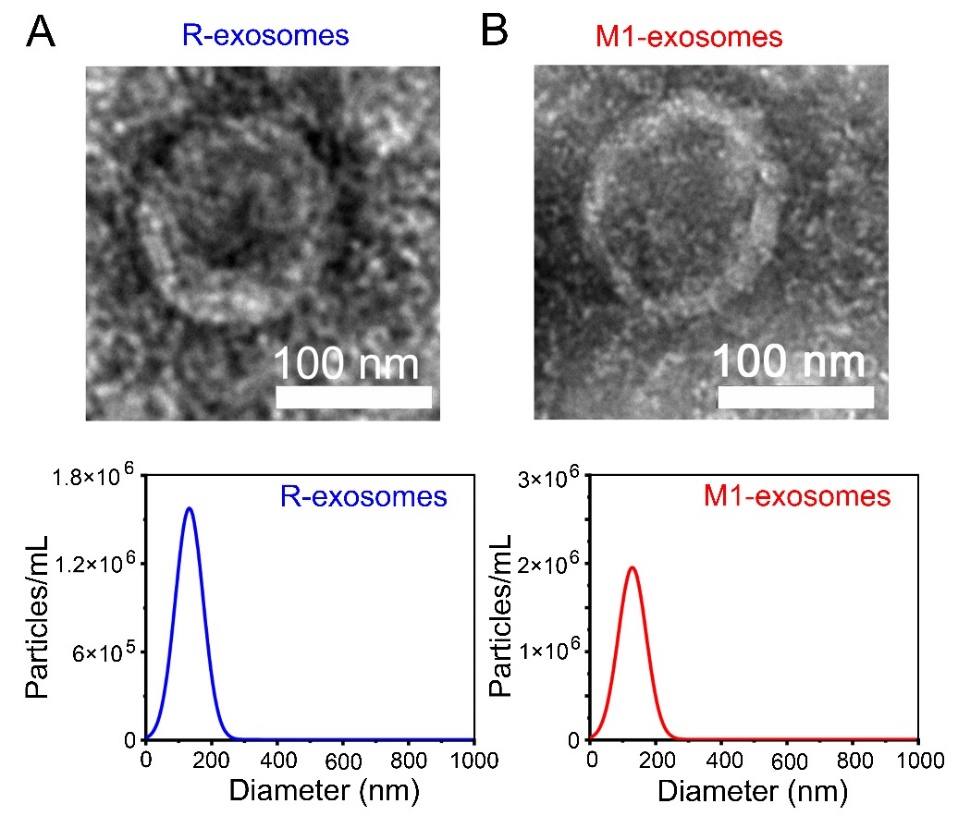


**Figure S6.** Transmission electron microscope (TEM) analysis and Nanoparticle tracking analysis of exosomes derived from RAW264.7 cells and M1 macrophages. M1 macrophages were induced by the stimulation of RAW264.7 cells with LPS at a concentration of 500 nM for 5 days.


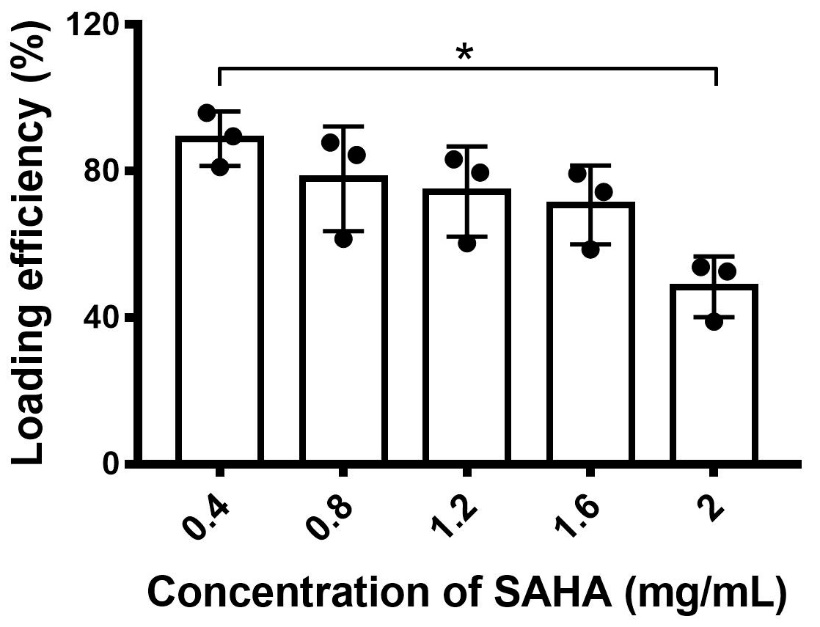


**Figure S7.** The loading efficiency of SUC at different concentrations of SAHA. SAHA was loaded into SUC at a serial of mass ratios: 1:0.4, 1:0.8, 1:1.2, 1:1.6, and 1:2. The data were represented as Mean ± SD, n=3. The statistical significance was calculated via one-way ANOVA with a Tukey post-hoc test. *P < 0.05.


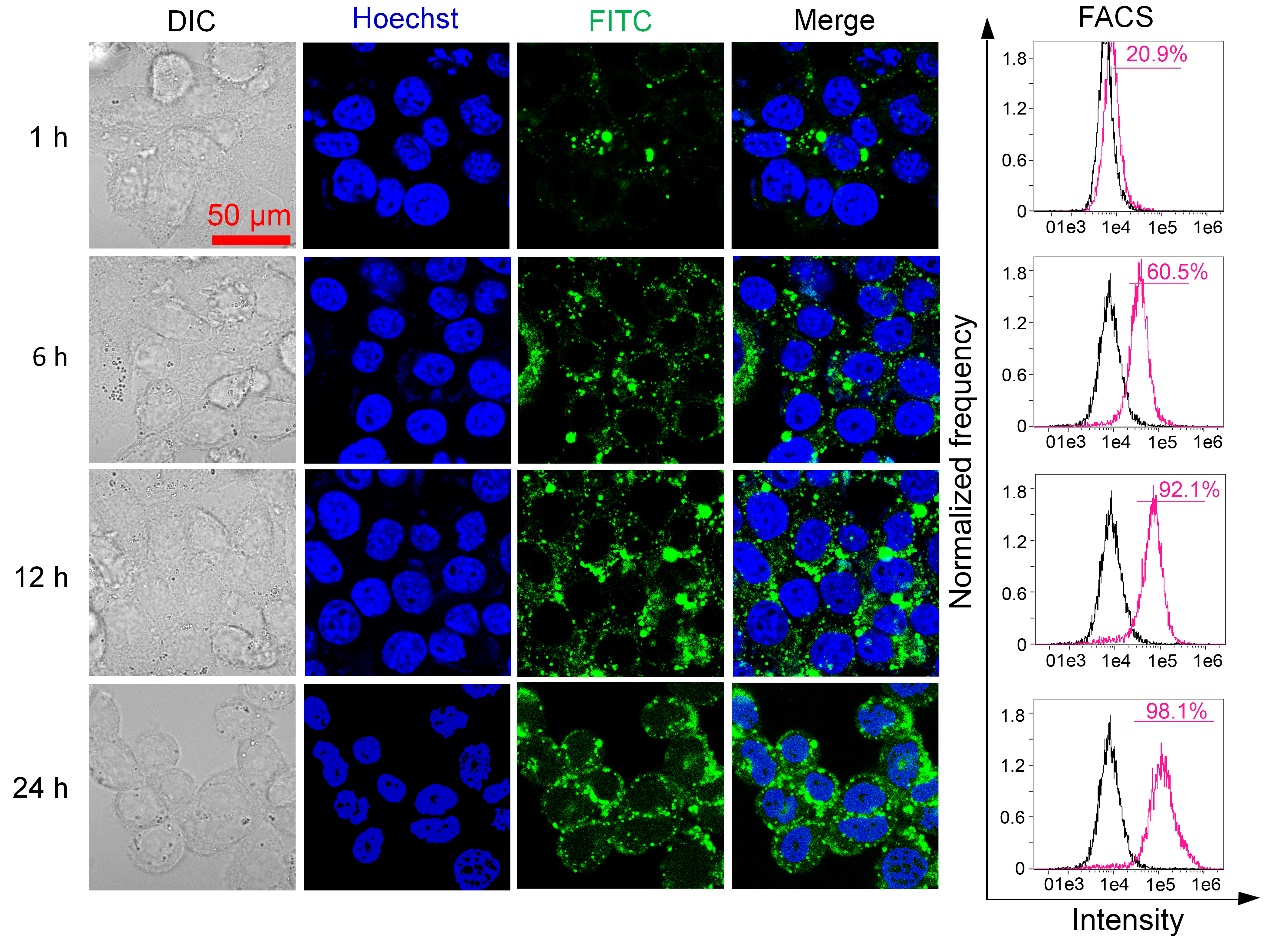


**Figure S8.** Time-dependent cellular uptake of EMS. LLCs were incubated with EM/SUC/FITC for 1, 6, 12, and 24 h, respectively.


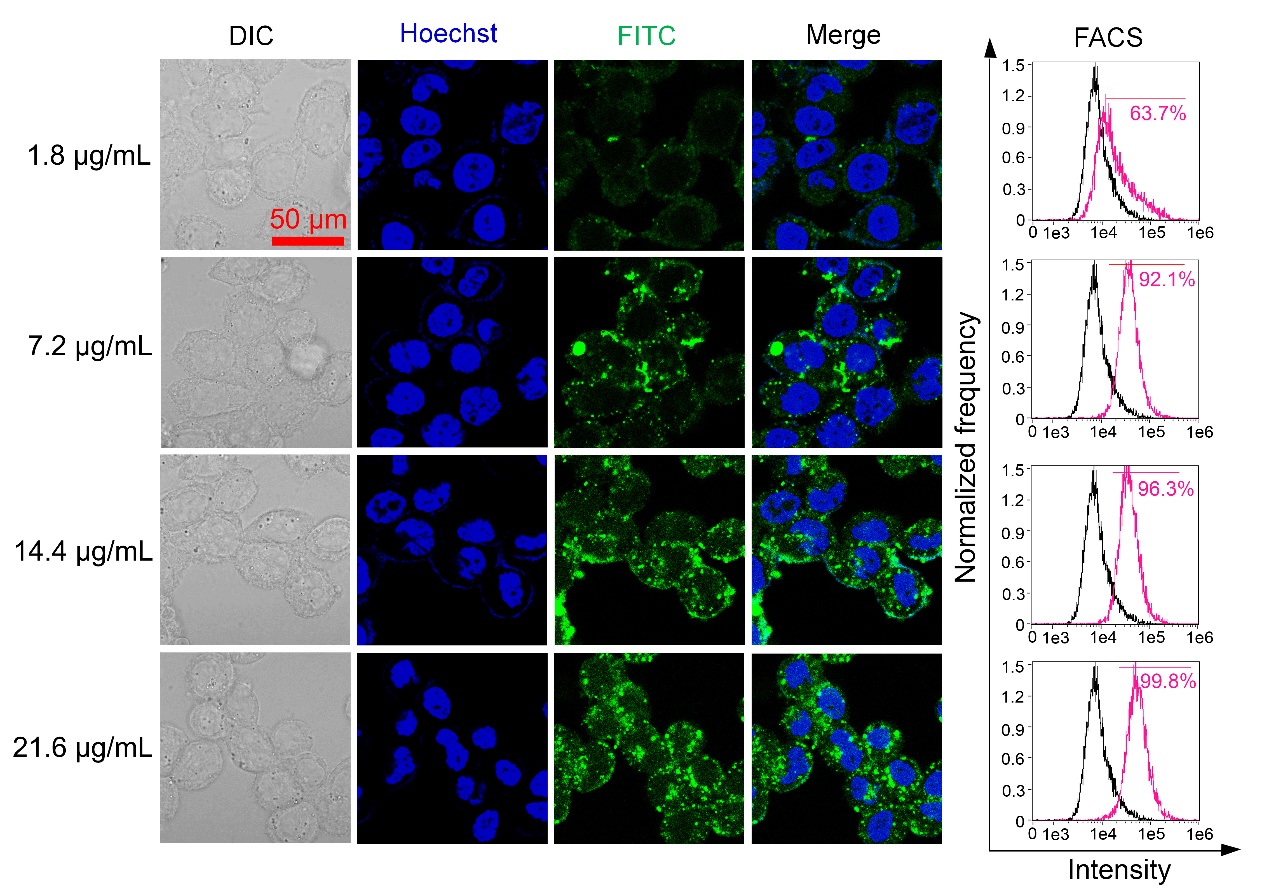


**Figure S9.** Dosage-dependent cellular uptake. LLCs were incubated with EM/SUC/FITC at different concentrations (1.8, 7.2, 14.4, and 21.6 μg/mL), respectively.


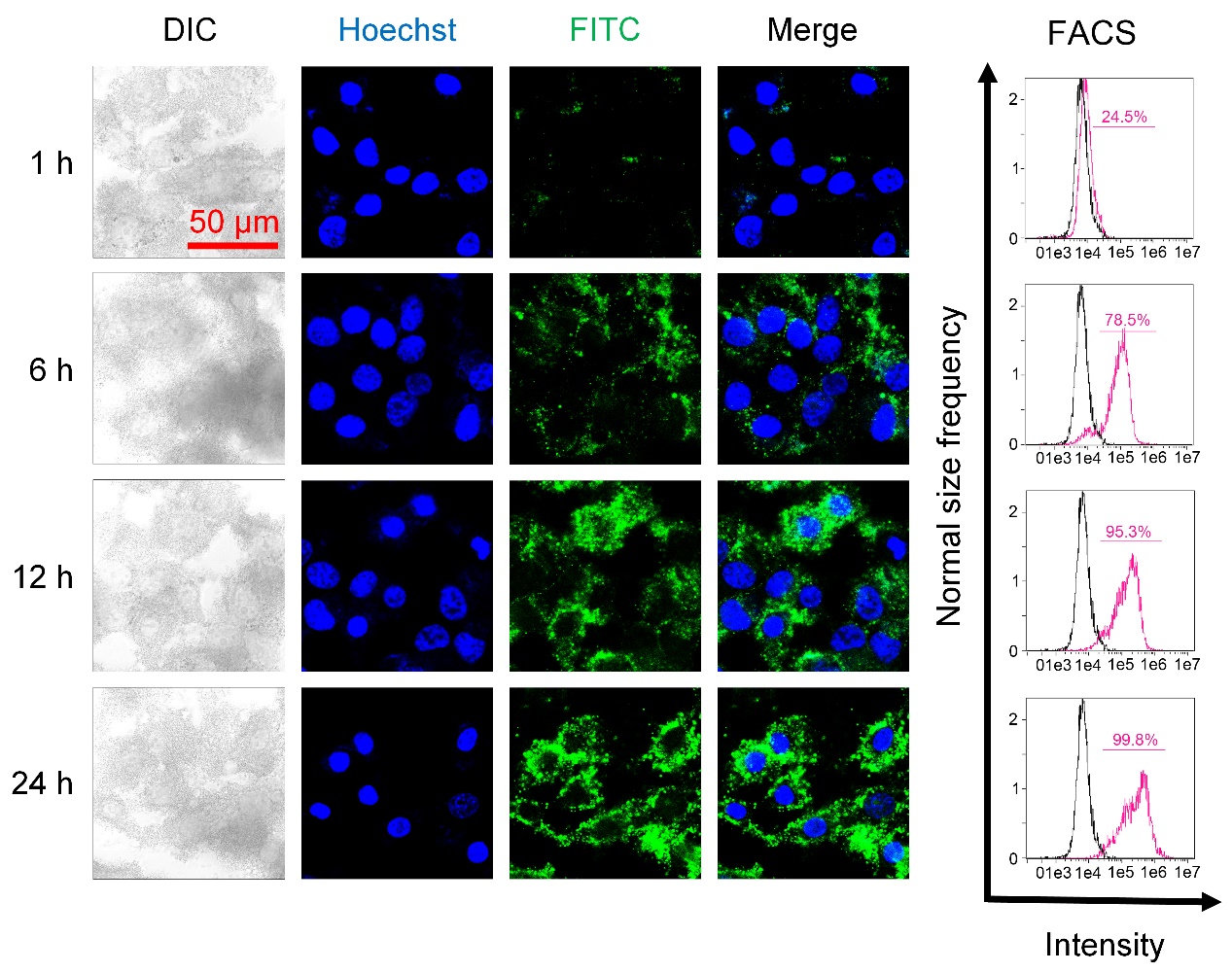


**Figure S10.** Time-dependent cellular uptake of EMS. A549 cells were incubated with EM/SUC/FITC for 1, 6, 12, and 24 h, respectively.


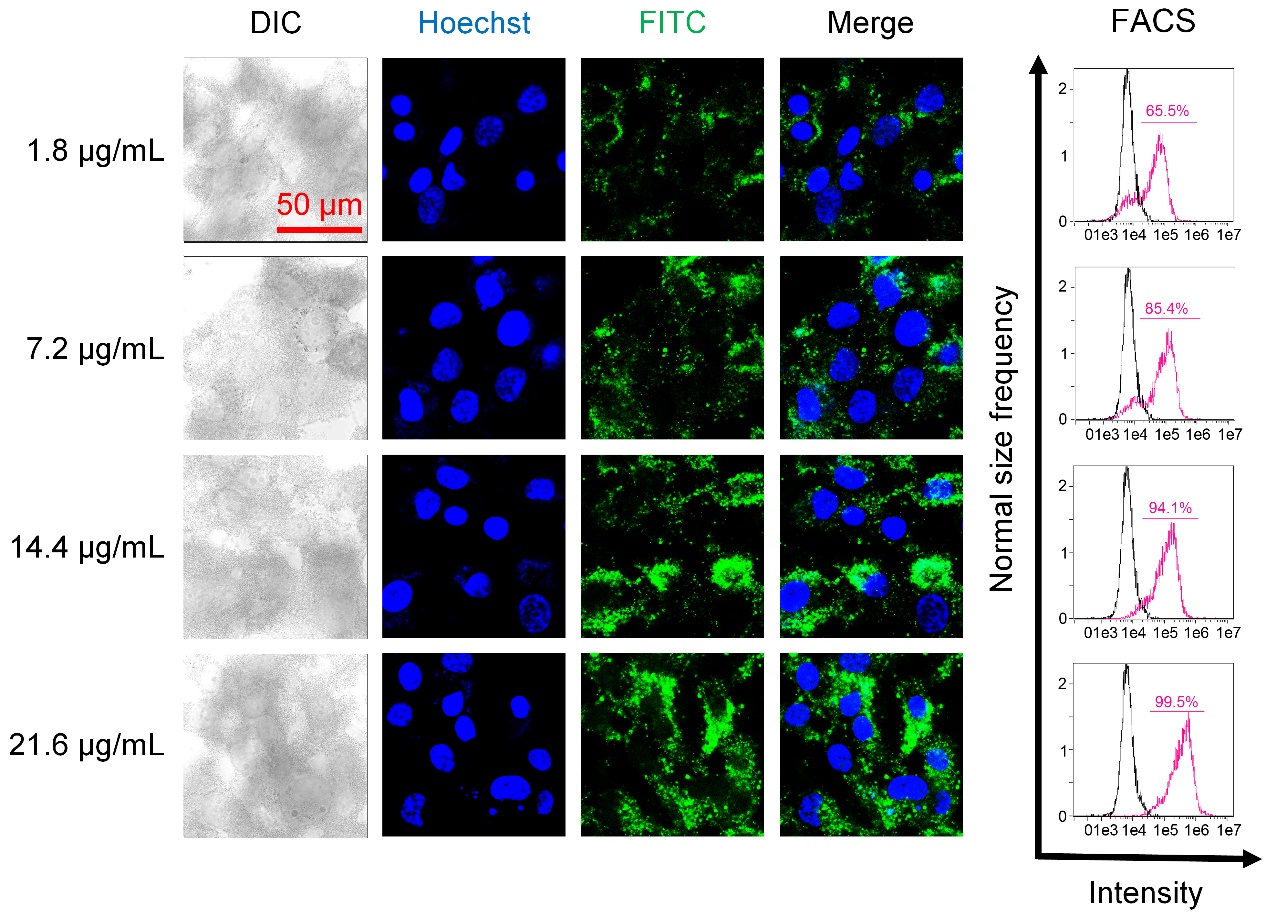


**Figure S11.** Dosage-dependent cellular uptake. A549 cells were incubated with EM/SUC/FITC at different concentrations (1.8, 7.2, 14.4, and 21.6 μg/mL), respectively.


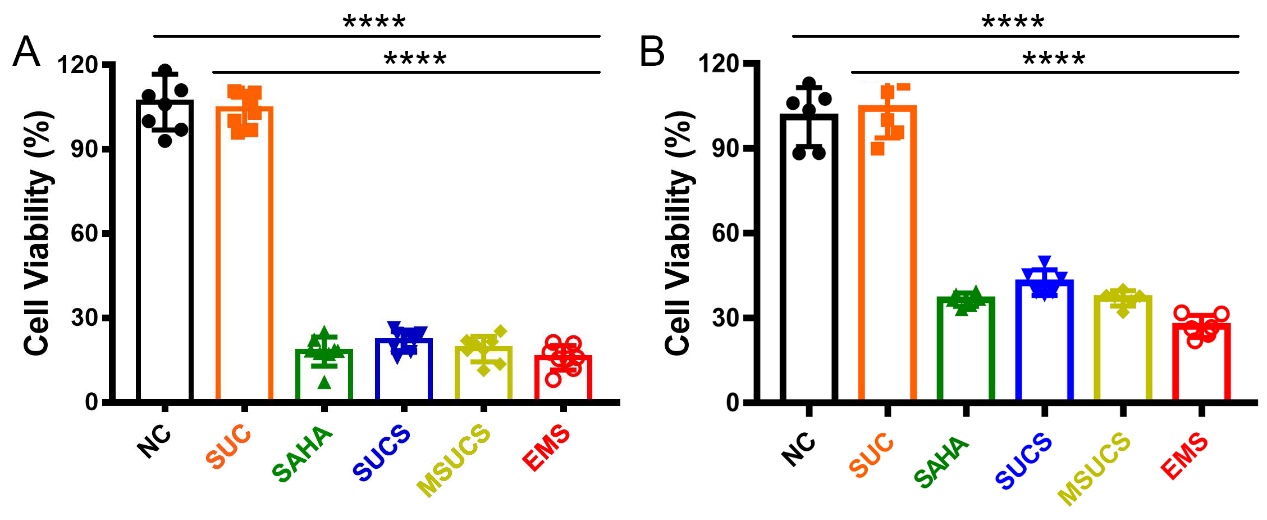


**Figure S12.** Cell viability after the treatment with different formulations. A) LLC cells. B) A549 cells. The dosage of SAHA was 8 μg/mL. The data was represented as mean ± SD. Statistical significance was calculated via one-way ANOVA with a Tukey post-hoc test.


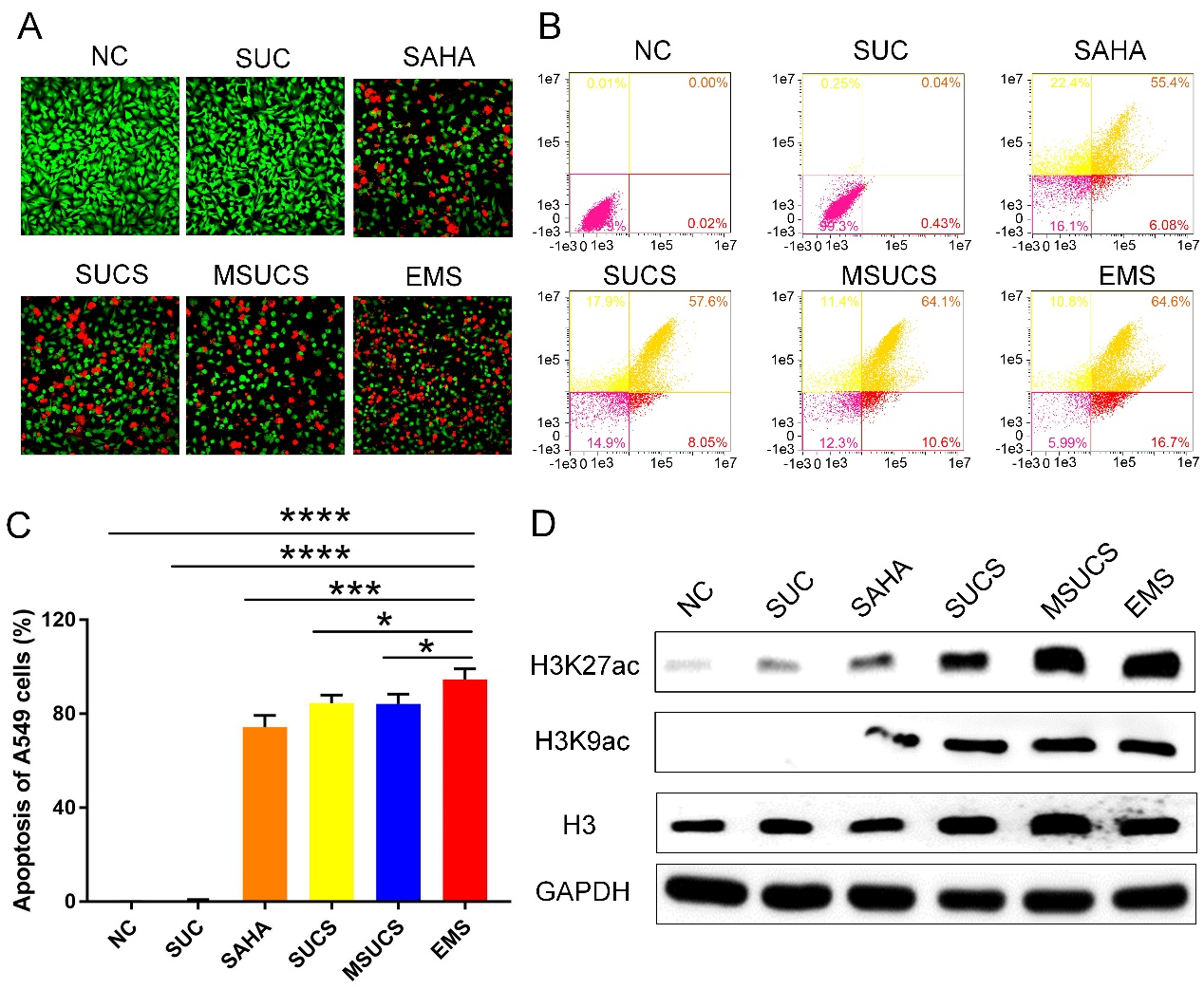


**Figure S13.** Different formulations induced epigenetic regulation. A) Live/Dead analysis of A549 cells treated with different formulations. B) Apoptosis of A549 cells treated with different formulations. C) The quantitative analysis of apoptosis of A549 cells (n=3). D) Western blotting analysis of A549 cells treated with different formulations. The data in C) was represented as mean ± SD. Statistical significance was calculated via one-way ANOVA with a Tukey post-hoc test. *p<0.05, ***p<0.001, ****p<0.0001.


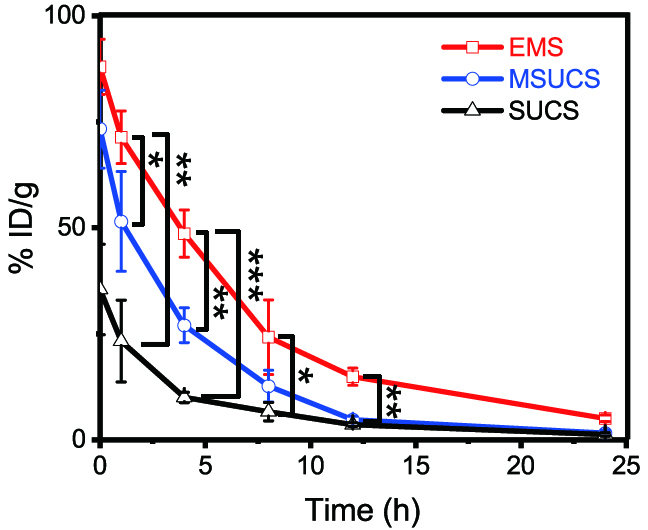


**Figure S14.** Circulation lifetime of different nanoparticles. EMS was labeled with DiD and administrated to the mice through tail intravenous injection. The blood was collected at different time points (0, 1, 4, 8, 12, and 24 h), respectively. The fluorescence intensity was examined with a multifunctional microplate reader. The data was represented as mean ± SD. Statistical significance was calculated via one-way ANOVA with a Tukey post-hoc test. *p<0.05, **p<0.01, ****p<0.0001.
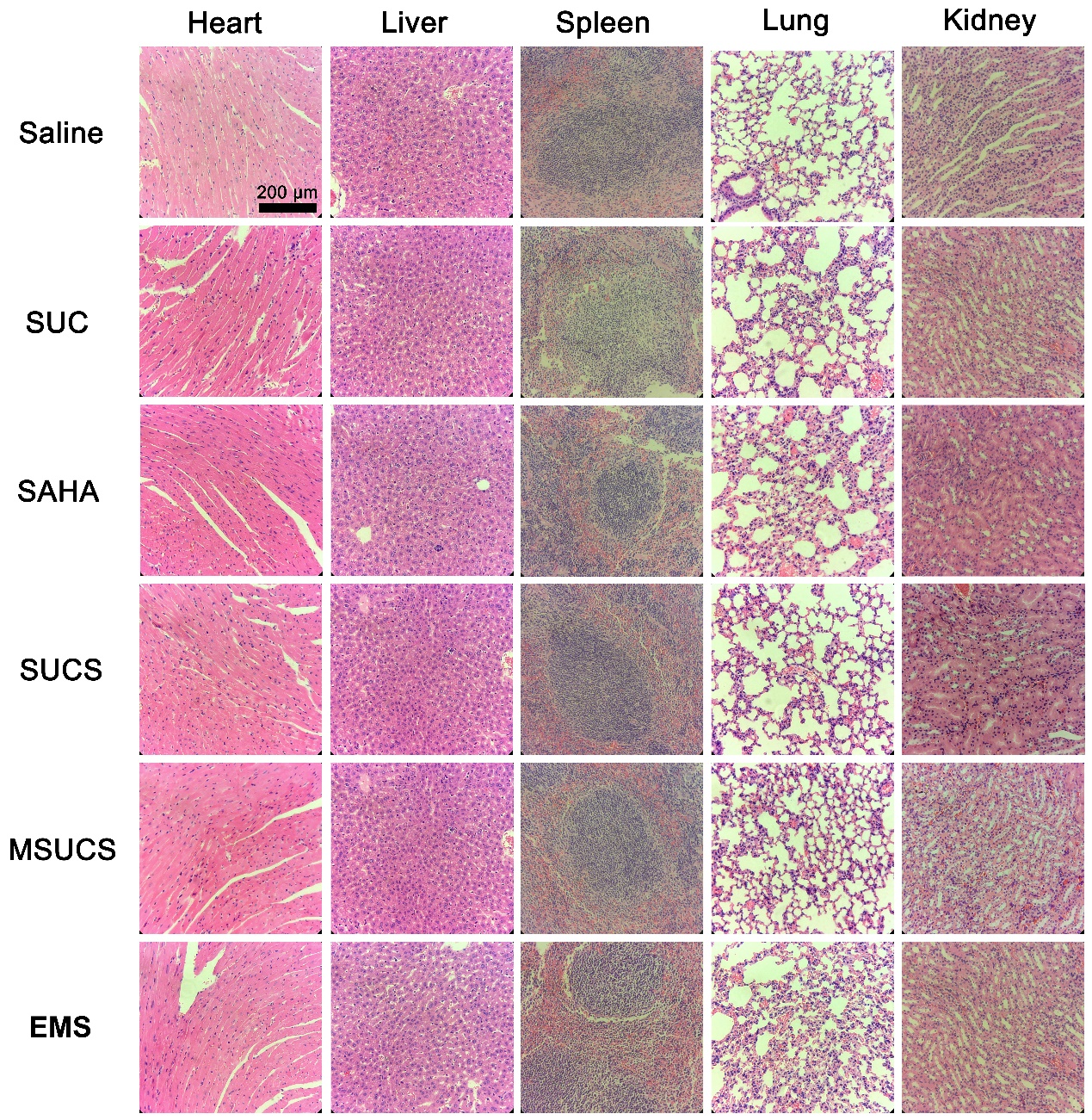


**Figure S15.** HE staining of the extracted organs. HE staining of the extracted organs. LLC-bearing mice were administrated with EMS every three days. After 15 days, the mice were sacrificed and the major organs were extracted and stained with HE. Saline, SAHA, SUC, SUCS, or MSUCS were used as control.
